# Activation of APOBEC3 cytidine deaminases and endogenous retroviruses is integrated by MUC1-C in NSCLC cells

**DOI:** 10.1038/s41420-025-02673-9

**Published:** 2025-08-08

**Authors:** Naoki Haratake, Shinkichi Takamori, Hideko Isozaki, Keisuke Shigeta, Chie Kikutake, Hiroki Ozawa, Atrayee Bhattacharya, Ayako Nakashoji, Mikita Suyama, Tomoyoshi Takenaka, Tomoharu Yoshizumi, Atsushi Osoegawa, Aaron N. Hata, Donald Kufe

**Affiliations:** 1 Department of Medical Oncology, Dana-Farber Cancer Institute, Harvard Medical School, Boston, MA USA; 2 Department of Thoracic and Breast Surgery, Oita University Faculty of Medicine, Oita, Japan; 3 Department of Medicine, Massachusetts General Hospital, Harvard Medical School, Boston, MA USA; 4 Division of Bioinformatics, Medical Institute of Bioregulation, Kyushu University, Fukuoka, Japan; 5 Department of Surgery and Science, Graduate School of Medical Sciences, Kyushu University, Fukuoka, Japan

**Keywords:** Cancer genomics, Cancer therapeutic resistance

## Abstract

The *APOBEC3 (A3)* genes encoding cytidine deaminases evolved in mammals to restrict retroviral replication. The *MUC1* gene appeared in mammals to protect barrier tissues from viral infections. There is no known involvement of the *MUC1* encoded MUC1-C/M1C protein in the regulation of A3s. We found that induction of MUC1-C in NSCLC cells treated with EGFR inhibitors integrates activation of an inflammatory memory response with the type I interferon (IFN) STAT1/STAT2/IRF9 (U-ISGF3) pathway. In turn, MUC1-C drives expression of A3 genes by activating their U-ISGF3-stimulated response elements (ISREs). We also report that MUC1-C-mediated induction of type II IFN STAT1 homodimer (U-GAF) complexes and the gamma-associated signaling (GAS) pathway drives human endogenous retrovirus HERV-K102/K108 expression. Our results in NSCLC cell line and patient-derived models further demonstrate that MUC1-C activates A3 and HERV-K expression by a common MUC1-C→STAT1 auto-inductive pathway. These previously unrecognized findings demonstrate that a MUC1-C-driven inflammatory pathway coordinates activation of APOBEC3 and HERV-K expression.

## Introduction

The apolipoprotein B mRNA-editing catalytic 3 (APOBEC3; A3A, A3B, A3C, A3D, A3F, A3G and A3H) enzymes evolved in mammals to restrict replication of endogenous retroviruses [1, 2]. The A3s catalyze deamidation of cytidine to uridine with resulting C→T or C→G substitutions at TpC motifs [3, 4]. Mutational signatures across the genomes of cancer cells attributed to A3s have been linked to tumor heterogeneity and treatment resistance [5–10]. Induction of A3A in non-small cell lung cancer (NSCLC) cells treated with receptor tyrosine kinase inhibitors (TKIs) promotes genomic instability and the development of drug resistance that contributes to disease relapse [11–13]. A3B is also induced in NSCLC cells during acquired resistance to EGFR TKIs [14, 15]. Dysregulation of A3A and A3B drives mutagenic signatures in cancer cells [5–10]. A3C and A3D promote DNA replication stress resistance [16]. Moreover, A3G protects cells from DNA damage [17] and confers a unique mutational signature [18]. These findings indicate that the A3s play distinct roles in cancer mutagenesis and progression. Remarkably, the mechanisms responsible for regulating expression of A3A, A3B and other APOBEC3 enzymes have largely remained unclear.

The *MUC1* gene evolved in mammals to provide barrier tissues, such as respiratory epithelia, with protection from viral infections and abiotic environmental insults [19–21]. *MUC1* encodes a transmembrane non-mucin MUC1-C/M1C subunit that is activated in barrier epithelia by loss of homeostasis [19–21]. As a result, MUC1-C induces inflammatory, proliferative and remodeling signaling pathways that contribute to wound healing [19–21]. These responses are in principle reversible with repair; whereas, prolonged activation of MUC1-C by chronic inflammation contributes to established epigenetic alterations and cancer progression [20, 21]. Like A3A and A3B [11–15], MUC1-C is activated in NSCLC EGFR mutant cells treated with TKIs [22]. MUC1-C functions as a common effector of acquired NSCLC cell resistance to TKIs associated with pleotropic mechanisms, including the epithelial mesenchymal transition (EMT), *MET* amplification and secondary EGFR(T790M/C797S) mutation [22]. MUC1-C binds directly to STAT1 and regulates STAT1 target genes, including *MUC1* in an inflammatory auto-inductive pathway [23]. MUC1-C has also been linked to STAT1-mediated activation of the interferon (IFN) type I and II pathways and DNA damage resistance in other types of cancer cells [24–26]. We therefore reasoned that MUC1-C→STAT1 signaling could contribute to resistance of NSCLC cells to TKIs by activating pathways that promote genomic instability.

Dysregulation of A3s has been linked to treatment resistance; however, there is no known relationship between MUC1-C signaling and A3 expression in cancer cells. The present work demonstrates that MUC1-C-dependent activation of the type I IFN STAT1/STAT2/IRF9 (U-ISGF3) pathway drives induction of A3A, A3G and other A3 genes. The A3s evolved to restrict endogenous retroviral expression [1, 2]. Our results further demonstrate that MUC1-C activates the type II IFN STAT1 homodimer (U-GAF) and gamma-associated signaling (GAS) pathway in driving human retroviral K (HERV-K) expression. STAT1 is an indispensable component of ISRE- and GAS-driven IFN stimulated genes (ISGs) that in turn amplify the type I and II IFN pathways [27, 28]. We report that MUC1-C integrates induction of a STAT1 inflammatory pathway with activation of A3 and HERV-K expression.

## Results

### MUC1-C regulates A3A expression in NSCLC cells

**Fig. 1:**
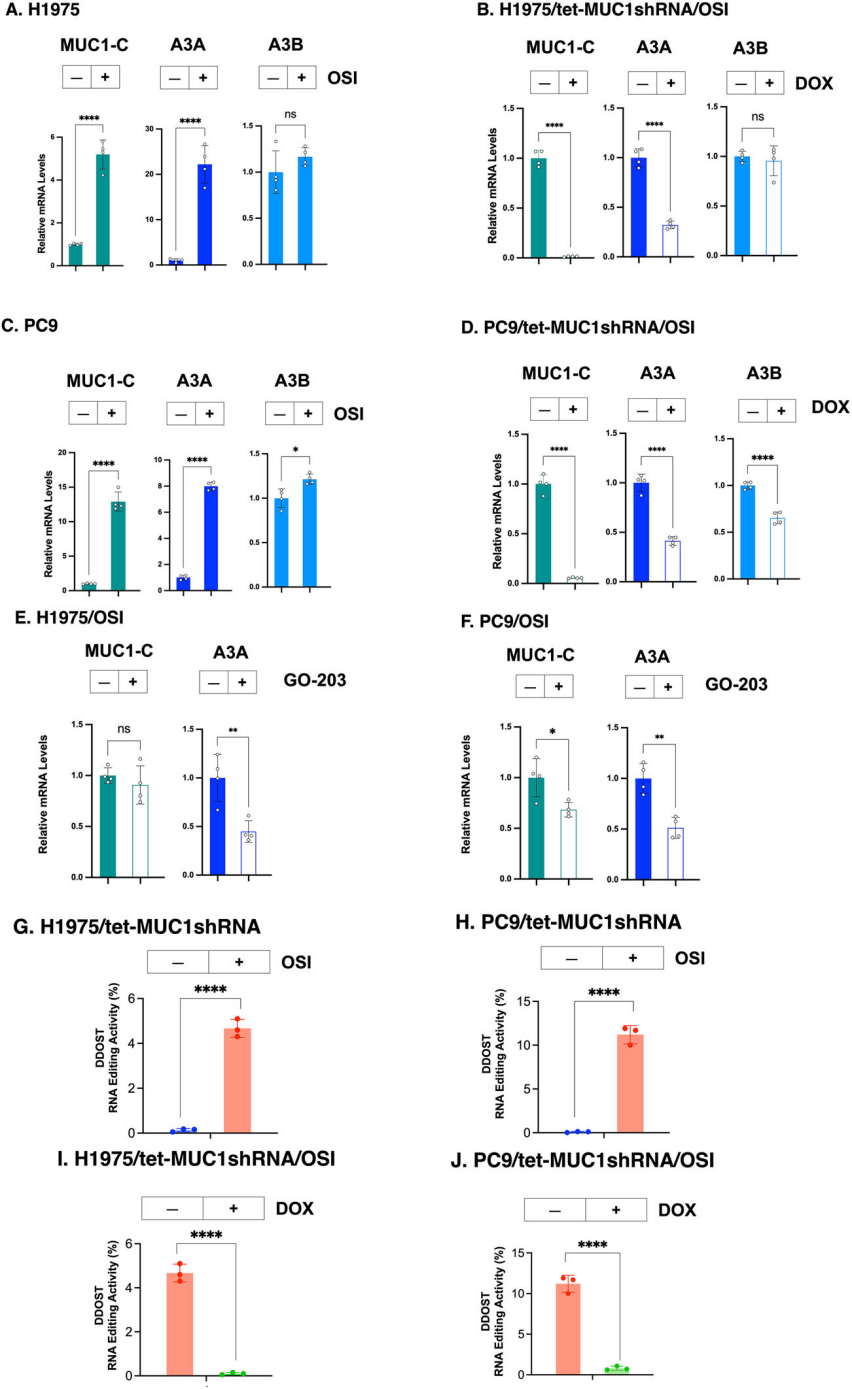
A3A expression is upregulated in NSCLC cells by a MUC1-C-dependent mechanism. **A** H1975 cells treated with vehicle or 1 μM OSI for 3 days were analyzed for the indicated transcripts by qRT-PCR using primers listed in Supplementary Table S1. The results (mean ± SD of 4 determinations) are expressed as relative levels compared to that obtained for vehicle-treated cells (assigned a value of 1). **B** H1975/tet-MUC1shRNA treated with 1 μM OSI for 3 days and vehicle or DOX for 7 days were analyzed for the indicated transcripts by qRT-PCR. The results (mean ± SD of 4 determinations) are expressed as relative levels compared to that obtained for vehicle-treated DTPs (assigned a value of 1). **C** PC9 cells treated with 1 μM OSI for 3 days were analyzed for the indicated transcripts by qRT-PCR. The results (mean ± SD of 4 determinations) are expressed as relative levels compared to that obtained for untreated cells (assigned a value of 1). **D** PC9/tet-MUC1shRNA treated with 1 μM OSI for 3 days and vehicle or DOX for 7 days were analyzed for the indicated transcripts by qRT-PCR. The results (mean ± SD of 4 determinations) are expressed as relative levels compared to that obtained for vehicle-treated cells (assigned a value of 1). H1975 (**E**) and PC9 (**F**) cells treated with 1 μM OSI and vehicle or 1 μM GO-203 for 3 days were analyzed for the indicated transcripts by qRT-PCR. The results (mean ± SD of 4 determinations) are expressed as relative levels compared to that obtained for vehicle-treated cells (assigned a value of 1). H1975 (**G**) and PC9 (**H**) cells treated with 1 μM OSI for 3 days were analyzed for A3A editing activity. H1975/tet-MUC1shRNA (**I**) and PC9/tet-MUC1shRNA (**J**) cells treated with 1 μM OSI for 3 days and vehicle or DOX for 7 days were analyzed for A3A editing activity. The results (mean ± SD of 3 determinations) are expressed as DDOST RNA editing activity (% of DDOST 558 C→U).

A3A and A3B expression is upregulated in NSCLC TKI-resistant cells that promote relapse and progression [13–15, 29]. MUC1-C promotes survival of NSCLC TKI-treated cells [22]. We therefore asked if MUC1-C plays a role in regulating A3s. We initially found that expression of MUC1-C and A3A, but not A3B, is significantly increased in H1975 NSCLC cells treated with the EGFR TKI osimertinib (OSI) (Figs. 1A and S1A).

Interestingly, silencing expression of the MUC1-C ~25 kDa glycosylated and 17 kDa unglycosylated proteins in H1975 cells [30] (Fig. S1A, S1B) suppressed OSI-induced upregulation of A3A expression (Fig. 1B). Silencing MUC1-C with an additional MUC1shRNA#2 (Fig. S1C) confirmed suppression of OSI-mediated A3A induction (Fig. S1D). Analysis of PC9/EGFR(19del) NSCLC cells treated with OSI further identified upregulation of MUC1-C in association with increases in A3A and, to a lesser extent, A3B expression (Figs. 1C and S1E). As found in H1975 cells, we confirmed that A3A is regulated in PC9 cells by a MUC1-C-dependent mechanism (Figs. 1D and S1F). The transmembrane MUC1-C protein consists of a 72 aa intrinsically disordered cytoplasmic domain (MUC1-CD) [31]. MUC1-CD includes a CQC motif necessary for MUC1-C homodimerization, nuclear import and function [31]. Treatment with the GO-203 inhibitor, which targets the CQC motif [22], suppressed induction of A3A transcripts in OSI-treated H1975 (Fig. 1E) and PC9 (Fig. 1F) cells. Of note, A3A protein levels in H1975 and PC9 cells were below detectability by immunoblotting.

To assess effects of MUC1-C on A3A enzymatic activity, we analyzed A3A RNA editing by digital PCR at UpC sites within a defined stem-loop hairpin motif [13]. Consistent with increases in A3A expression, C→U editing within the DDOST gene mRNA (DDOST 558 C→U) was substantially higher in OSI-treated (i) H1975 (Fig. 1G) and (ii) PC9 (Fig. 1H) cells. Significantly, silencing MUC1-C markedly suppressed the OSI-induced increases in C→U editing (Fig. 1I, J). These results indicated that MUC1-C drives A3A expression and activity in the response of H1975 and PC9 cells to TKI treatment.

### MUC1-C drives A3A expression in NSCLC cells by the type I IFN inflammatory pathway

**Fig. 2:**
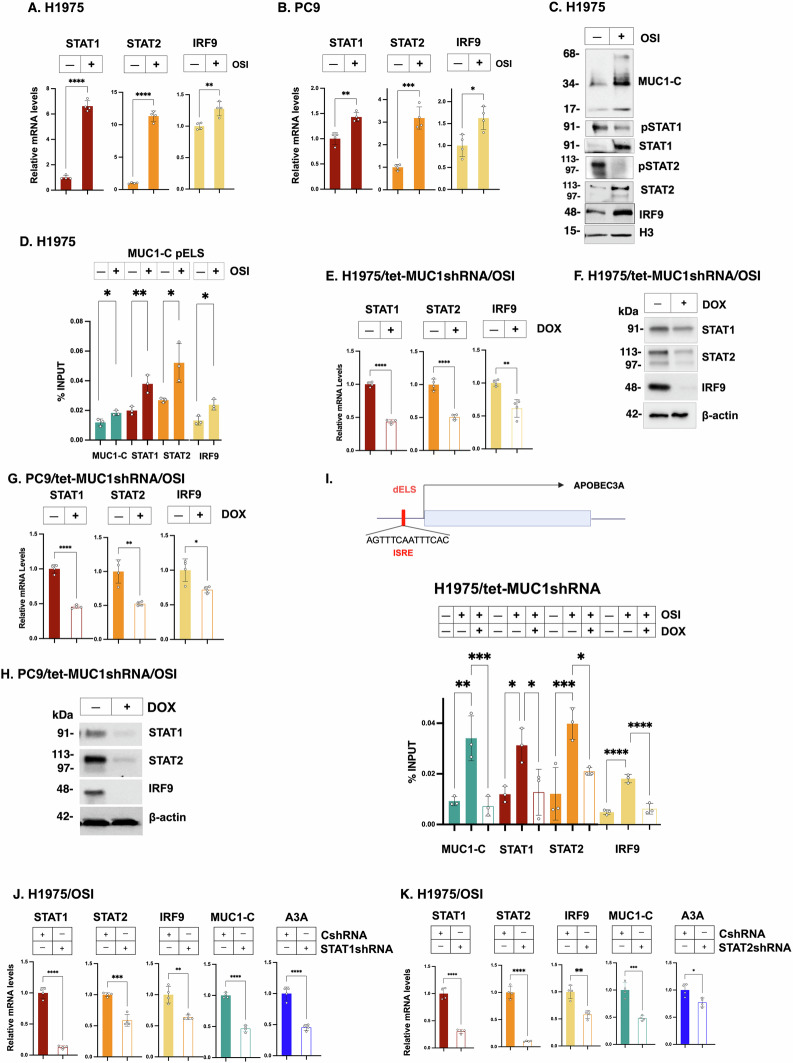
MUC1-C activates type I IFN signaling that induces A3A expression. H1975 (**A**) and PC9 (**B**) cells treated with 1 μM OSI for 3 days were analyzed for STAT1, STAT2 and IRF9 mRNA levels by qRT-PCR. The results (mean ± SD of 4 determinations) are expressed as relative levels compared to that obtained for control cells (assigned a value of 1). **C** Chromatin from H1975 cells treated with 1 μM OSI for 3 days was immunoblotted with antibodies against the indicated proteins. **D** Soluble chromatin from H1975 cells treated with 1 μM OSI for 3 days was precipitated with antibodies against MUC1-C, STAT1, STAT2 and IRF9. The DNA samples were amplified by qPCR with primers for the *MUC1* dELS region. The results (mean ± SD of 4 determinations) are expressed as % input. **E**, **F** H1975/tet-MUC1shRNA cells treated with 1 μM OSI for 3 days and vehicle or DOX for 7 days were analyzed for the indicated transcripts by qRT-PCR. The results (mean ± SD of four determinations) are expressed as relative levels compared to that obtained for vehicle-treated cells (assigned a value of 1) (**E**). Lysates were immunoblotted with antibodies against the indicated proteins (**F**). **G**, **H** PC9/tet-MUC1shRNA cells treated with 1 μM OSI for 3 days and vehicle or DOX for 7 days were analyzed for the indicated transcripts by qRT-PCR. The results (mean ± SD of four determinations) are expressed as relative levels compared to that obtained for vehicle-treated cells (assigned a value of 1) (**G**). Lysates were immunoblotted with antibodies against the indicated proteins (**H**). **I** Schema of *A3A* with highlighting of the pELS region containing a U-ISGF3 binding motif. Soluble chromatin from H1975/tet-MUC1shRNA cells treated with 1 μM OSI for 3 days and vehicle or DOX was precipitated with antibodies against MUC1-C, STAT1, STAT2 and IRF9. The DNA samples were amplified by qPCR with primers for the *A3A* pELS region. The results (mean ± SD of 4 determinations) are expressed as % input. H1975/CshRNA and H1975/STAT1shRNA (**J**) or H1975/STAT2shRNA (**K**) cells treated with 1 μM OSI for 3 days were analyzed for the indicated transcripts by qRT-PCR. The results (mean ± SD of 4 determinations) are expressed as relative levels compared to that obtained for CshRNA cells (assigned a value of 1).

MUC1-C forms a direct complex with STAT1 and regulates STAT1 target genes, including *MUC1 and STAT1* in an auto-inductive pathway [23–26]. Consistent with those findings, silencing MUC1-C in H1975 cells suppressed the REACTOME INTERFERON SIGNALING gene signature (Fig. S2A). Among these IFN signaling genes, we found that, in addition to STAT1 [23–26], MUC1-C drives expression of STAT2 and IRF9. In this regard, STAT1, STAT2 and IRF9 form (U-ISGF3) transcriptional complexes that activate ISGs with ISREs [32, 33]. We also found that MUC1-C, STAT1, STAT2 and IRF9 are induced in the response of H1975 (Figs. 2A and S2B) and PC9 (Fig. 2B and S2C) cells to OSI treatment. The MUC1-C 17 kDa protein is expressed in chromatin as monomers and higher order multimers [22, 34]. MUC1-C was upregulated in chromatin from OSI-treated H1975 cells in association with increases in unphosphorylated STAT1 (U-STAT1), U-STAT2 and IRF9 levels, indicating that MUC1-C activates the U-ISGF3 pathway (Fig. 2C).

The *MUC1* gene is activated at a pELS domain that functions as a STAT1-dependent inflammatory domain [23]. Here, analysis of the *MUC1* pELS-1 region in OSI-treated H1975 cells demonstrated increases in occupancy of MUC1-C and STAT1, as well as STAT2 and IRF9 (Fig. 2D), in support of involvement of the U-ISGF3 pathway. Upregulation of *MUC1* expression and occupancy of the *MUC1* pELS-1 by U-ISGF3 were associated with increases in chromatin accessibility in OSI-treated H1975 (Fig. S2D) and PC9 (Fig. S2E, F) cells. These results indicated that U-ISGF3 activates the pELS-1 inflammatory domain. We also found that silencing MUC1-C in H1975 (Fig. 2E, F) and PC9 (Fig. 2G, H) cells decreases expression of STAT1, STAT2 and IRF9. Treatment of H1975 (Fig. S2G) and PC9 (Fig. S2H) cells with GO-203 similarly downregulated STAT1, STAT2 and IRF9 expression, in support of a potential MUC1-C→U-ISGF3→ISRE pathway.

The *A3A* gene includes a distal enhancer-like signature (dELS) with a consensus U-ISGF3 binding ISRE motif (AGTTTCAATTTCAC) (Fig. 2I). Studies of H1975 cells treated with OSI identified significant increases in MUC1-C, STAT1, STAT2 and IRF9 occupancy on the *A3A* dELS that were suppressed by MUC1-C silencing (Fig. 2I). In support of these results, silencing STAT1 and STAT2 attenuated upregulation of A3A in H1975 (Fig. 2J, K) and PC9 (Fig. S2I, S2J) cells. Noteworthy is that, in addition to A3A, (i) silencing STAT1 decreased MUC1-C, STAT2 and IRF9 (Figs. 2J and S2I), and (ii) silencing STAT2 decreased MUC1-C, STAT1 and IRF9 (Figs. 2K and S2J) expression, consistent with intersection of MUC1-C, STAT1 and STAT2 signaling. These findings indicate that MUC1-C induces U-ISGF3 complexes that integrate activation of the *MUC1* pELS-1 inflammatory memory domain with A3A expression.

### Regulation of A3A is MUC1-C-dependent in patient-derived NSCLC cells

**Fig. 3:**
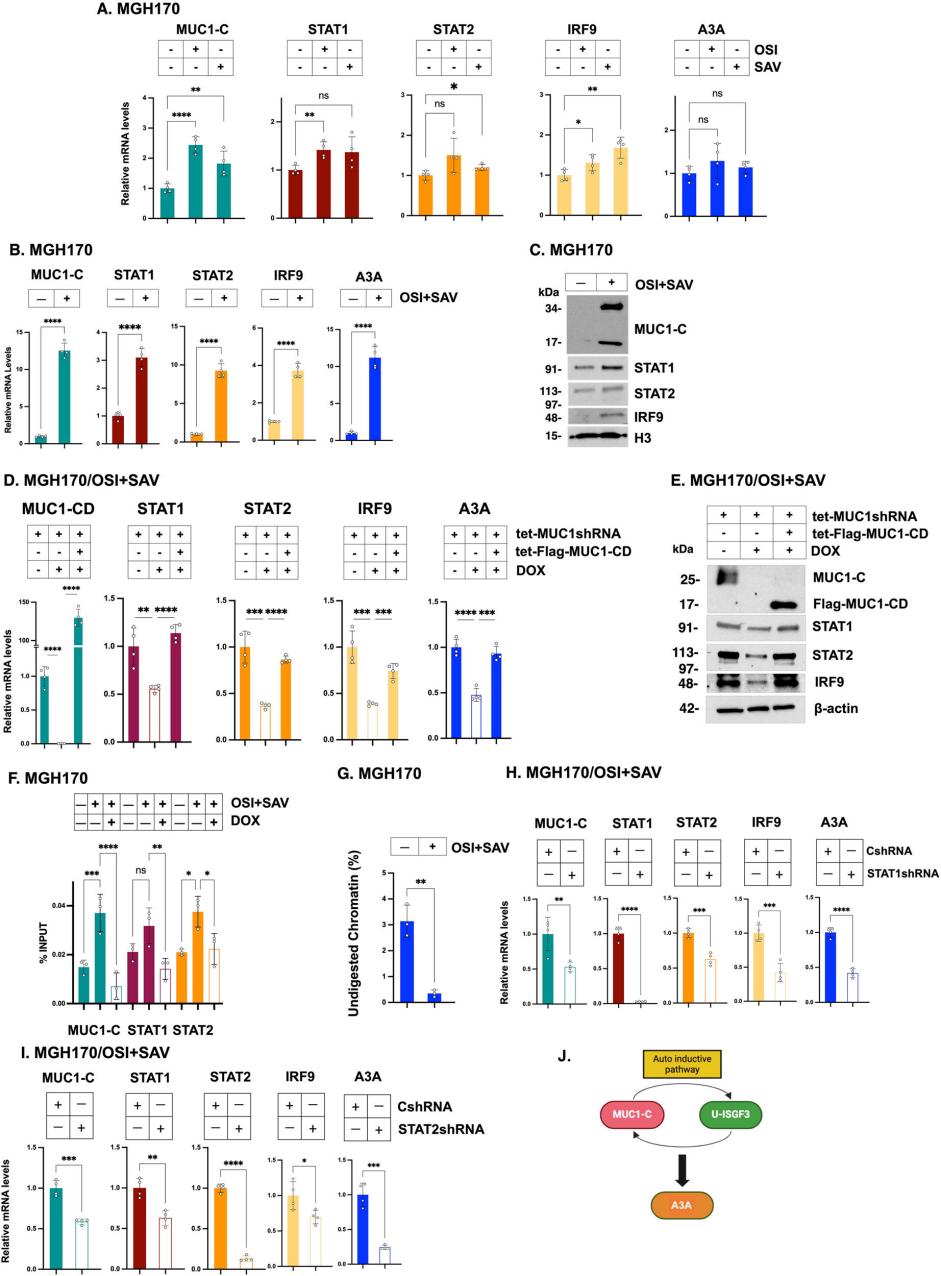
MUC1-C→type I IFN pathway regulates A3A expression. **A** MGH170 cells treated with 1 μM OSI or 1 μM SAV for 3 days were analyzed for the indicated transcripts by qRT-PCR. The results (mean ± SD of 4 determinations) are expressed as relative levels compared to that obtained for control cells (assigned a value of 1). **B** MGH170 cells treated with 1 μM OSI + 1 μM SAV for 3 days were analyzed for the indicated transcripts by qRT-PCR. The results (mean ± SD of 4 determinations) are expressed as relative levels compared to that obtained for control cells (assigned a value of 1). **C** Chromatin from MGH170 cells treated with 1 μM OSI + 1 μM SAV was immunoblotted with antibodies against the indicated proteins. **D**, **E** MGH170 cells expressing the designated vectors were treated with OSI + SAV for 3 days and vehicle or DOX for 7 days and analyzed for the indicated transcripts by qRT-PCR. The results (mean ± SD of 4 determinations) are expressed as relative levels compared to that obtained for vehicle-treated cells (assigned a value of 1) (**D**). Lysates were immunoblotted with antibodies against the indicated proteins (**E**). **F** Soluble chromatin from MGH170 cells treated with OSI + SAV was precipitated with antibodies against MUC1-C, STAT1, STAT2 and IRF9. The DNA samples were amplified by qPCR with primers for the *A3A* dELS1 region. The results (mean ± SD of 3 determinations) are expressed as % input. **G** Chromatin from MGH170 cells treated with OSI + SAV was analyzed for accessibility of the *A3A* dELS-1 region by nuclease digestion. The results (mean ± SD of 3 determinations) are expressed as % undigested chromatin. MGH170/CshRNA and MGH170/STAT1shRNA (**H**) or MGH170/STAT2shRNA (**I**) cells treated with OSI + SAV were analyzed for the indicated transcripts by qRT-PCR. The results (mean ± SD of 4 determinations) are expressed as relative levels compared to that obtained for CshRNA cells (assigned a value of 1). **J** Schema depicting the MUC1-C→U-ISGF3 auto-inductive pathway that regulates A3A expression.

To extend these studies, we focused on NSCLC MGH170-1D #2 (MGH170; *MET* amplification) cells obtained from a patient with acquired OSI resistance [35]. MGH170 cells treated with OSI exhibited increases in MUC1-C, STAT1 and IRF9 mRNA levels, whereas the upregulation of STAT2 and A3A was not significant (Fig. 3A). In this regard, OSI resistance of MGH170 cells is conferred by MUC1-C-driven MET activation [22]. MGH170 cells treated with the MET inhibitor savolitinib (SAV) also responded with significant increases in MUC1-C, STAT2, and IRF9, but not STAT1 or A3A, expression (Fig. 3A). These results were thus in contrast to what was found in OSI-treated H1975 and PC9 cells; that is, upregulation of MUC1-C, STAT1, STAT2 and IRF9 in driving A3A expression.

Studies of TKI resistant MGH170 cells growing in culture and as tumor xenografts have demonstrated that targeting MUC1-C is effective in reversing the resistant phenotype [22]. Along these lines and compared to OSI and SAV alone, MGH170 cells treated with OSI + SAV exhibited marked increases in (i) MUC1-C, STAT1, STAT2, IRF9 and A3A transcripts (Fig. 3B), and (ii) MUC1-C, STAT1, STAT2 and IRF9 proteins in chromatin (Fig. 3C). Furthermore, silencing MUC1-C in OSI + SAV-treated MGH170 cells decreased expression of STAT1, STAT2, IRF9 and A3A, which was rescued by the Flag-tagged MUC1-C cytoplasmic domain (MUC1-CD) detectable as a 17 kDa protein (Fig. 3D, E). Targeting MUC1-C in MGH170 cells with GO-203 also decreased STAT1, STAT2, IRF9 and A3A expression (Fig. S3A). By contrast, GO-203 had limited effects on MUC1-C mRNA levels (Fig. S3A), consistent with the function of this inhibitor in blocking MUC1-C downstream signaling pathways.

Analysis of the *A3A* pELS in OSI + SAV-treated MGH170 cells uncovered increases in (i) MUC1-C, STAT1, STAT2 and IRF9 occupancy (Fig. 3F), and (ii) chromatin accessibility (Fig. 3G). In addition, we confirmed that induction of A3A in MGH170 cells is STAT1- and STAT2-dependent (Fig. 3H, I), further supporting a MUC1-C → U-ISGF3 auto-inductive pathway that drives MUC1-C and A3A expression (Fig. 3J). MUC1 associates with poor clinical outcomes in patients with NSCLCs [22]. Of interest in this regard, upregulation of A3A in NSCLC tumors associates with a significant decrease in patient overall survival (OS) (Fig. S3B). We also found that upregulation of A3B, A3C, A3D, A3G and A3H, but not A3F, in NSCLCs is associated with decreases in OS (Fig. S3C, S3D). Given these findings, it was intriguing to speculate that MUC1-C might play a role in regulating other A3s that contribute to NSCLC progression.

### MUC1-C regulates multiple members of the A3 family

**Fig. 4:**
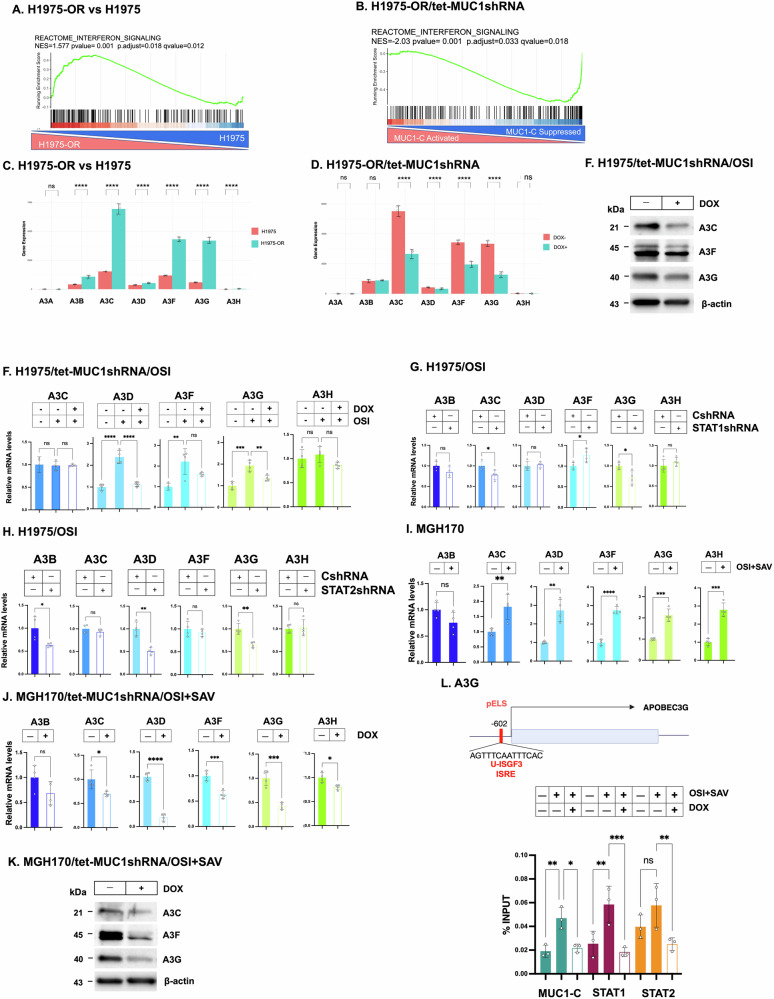
MUC1-C→type I IFN signaling regulates A3G and other A3s. GSEA of RNA-seq data from H1975-OR vs H1975 cells (**A**) and for H1975-OR/tet-MUC1shRNA cells treated with vehicle or DOX for 7 days (**B**) using the REACTOME INTERFERON SIGNALING gene signature. **C** Analysis of RNA-seq data from H1975 and H1975-OR cells for levels of A3 transcripts. **D** Analysis of RNA-seq data from H1975-OR/tet-MUC1shRNA cells treated with vehicle or DOX for 7 days for levels of A3 transcripts. The results are expressed as the mean ± SD of 3 biologically independent samples. **E** Lysates from H1975/tet-MUC1shRNA cells treated with 1 μM OSI for 3 days and vehicle or DOX for 7 days were immunoblotted with antibodies against the indicated proteins. **F** H1975/tet-MUC1shRNA cells treated with 1 μM OSI for 3 days and vehicle or DOX for 7 days were analyzed for the indicated transcripts by qRT-PCR. The results (mean ± SD of 4 determinations) are expressed as relative levels compared to that obtained for control cells (assigned a value of 1). H1975/CshRNA, H1975/STAT1shRNA (**G**) and H1975/STAT2shRNA (**H**) cells treated with 1 μM OSI for 3 days were analyzed for the indicated transcripts by qRT-PCR. The results (mean ± SD of 4 determinations) are expressed as relative levels compared to that obtained for CshRNA cells (assigned a value of 1). **I** MGH170 cells treated with OSI + SAV were analyzed for the indicated transcripts by qRT-PCR. The results (mean ± SD of 4 determinations) are expressed as relative levels compared to that obtained for control cells (assigned a value of 1). **J** MGH170/tet-MUC1shRNA treated with OSI + SAV for 3 days and vehicle or DOX for 7 days were analyzed for the indicated transcripts by qRT-PCR. The results (mean ± SD of 4 determinations) are expressed as relative levels compared to that obtained for vehicle-treated cells (assigned a value of 1). **K** Lysates from MGH170/tet-MUC1shRNA cells treated with OSI + SAV for 3 days and vehicle or DOX for 7 days were immunoblotted with antibodies against the indicated proteins. **L** Schema of *A3G* with highlighting of the pELS region containing a U-ISGF3 binding motif. Soluble chromatin from MGH170/tet-MUC1shRNA cells treated with OSI + SAV for 2 days and vehicle or DOX was precipitated with antibodies against MUC1-C, STAT1 and STAT2. The DNA samples were amplified by qPCR with primers for the *A3G* pELS region. The results (mean ± SD of 4 determinations) are expressed as % input.

We next asked if MUC1-C regulates A3A, A3B and other A3s in NSCLC cells with stable TKI resistance. H1975 cells were maintained in the presence of increasing OSI concentrations for 3 months for selection of an OSI resistant (OR) H1975-OR cell phenotype [22]. Analysis of RNA-seq data from H1975-OR vs H1975 cells demonstrated upregulation of the REACTOME INTERFERON SIGNALING gene signature (Fig. 4A). Moreover, silencing MUC1-C in H1975-OR cells was associated with suppression of the signature and downregulation of STAT1, STAT2 and IRF9 expression (Fig. 4B).

We found that H1975-OR cells express low levels of A3A and, by contrast, significant upregulation of A3B, A3C, A3D, A3F and A3G (Figs. 4C and S4A). Of these A3s, silencing MUC1-C in H1975-OR cells suppressed (i) A3C, A3F and A3G transcripts (Fig. 4D) and (ii) A3C and A3G protein levels (Fig. 4E). Analysis of H1975 cells treated with OSI further demonstrated significant increases in A3D, A3F and A3G, but not A3C or A3H, transcripts (Fig. 4F). Targeting MUC1-C decreased A3G expression (Fig. 4F), which like A3A, contributes to cancer mutagenesis [18]. Induction of A3G expression was also STAT1- and STAT2-dependent (Fig. 4G, H). Studies of PC9 cells similarly demonstrated significant upregulation of A3F, A3G and A3H (Fig. S4B). Here again, expression of A3G was dependent on MUC1-C (Fig. S4B), STAT1 (Fig. S4C) and STAT2 (Fig. S4D).

As additional evidence for MUC1-C→U-ISGF3 regulation of multiple A3s, MGH170 cells treated with OSI + SAV exhibited significant induction of A3C, A3D, A3F, A3G and A3H (Fig. 4I) by MUC1-C- (Figs. 4J and S4E), STAT1- (Fig. S4F) and STAT2- (Fig. S4G) dependent pathways. By extension, silencing MUC1-C also suppressed A3C A3F and A3G protein levels (Fig. 4K). We focused here on A3G, based on the demonstration that, like A3A, it is regulated by MUC1-C, STAT1 and STAT2. Along these lines, the *A3G* pELS region includes a consensus U-ISGF3 ISRE motif (AGTTTCAATTTCAC) (Fig. 4L). As shown for *A3A*, we found that the *A3G* pELS is occupied by MUC1-C, STAT1, STAT2 and IRF9 (Fig. 4L). Furthermore, silencing MUC1-C decreased STAT1, STAT2 and IRF9 occupancy (Fig. 4L). These findings indicate that, like *A3A*, MUC1-C→U-ISGF3→ISRE signaling regulates induction of the *A3G* gene.

### MUC1-C regulates HERV expression in NSCLC cells

**Fig. 5:**
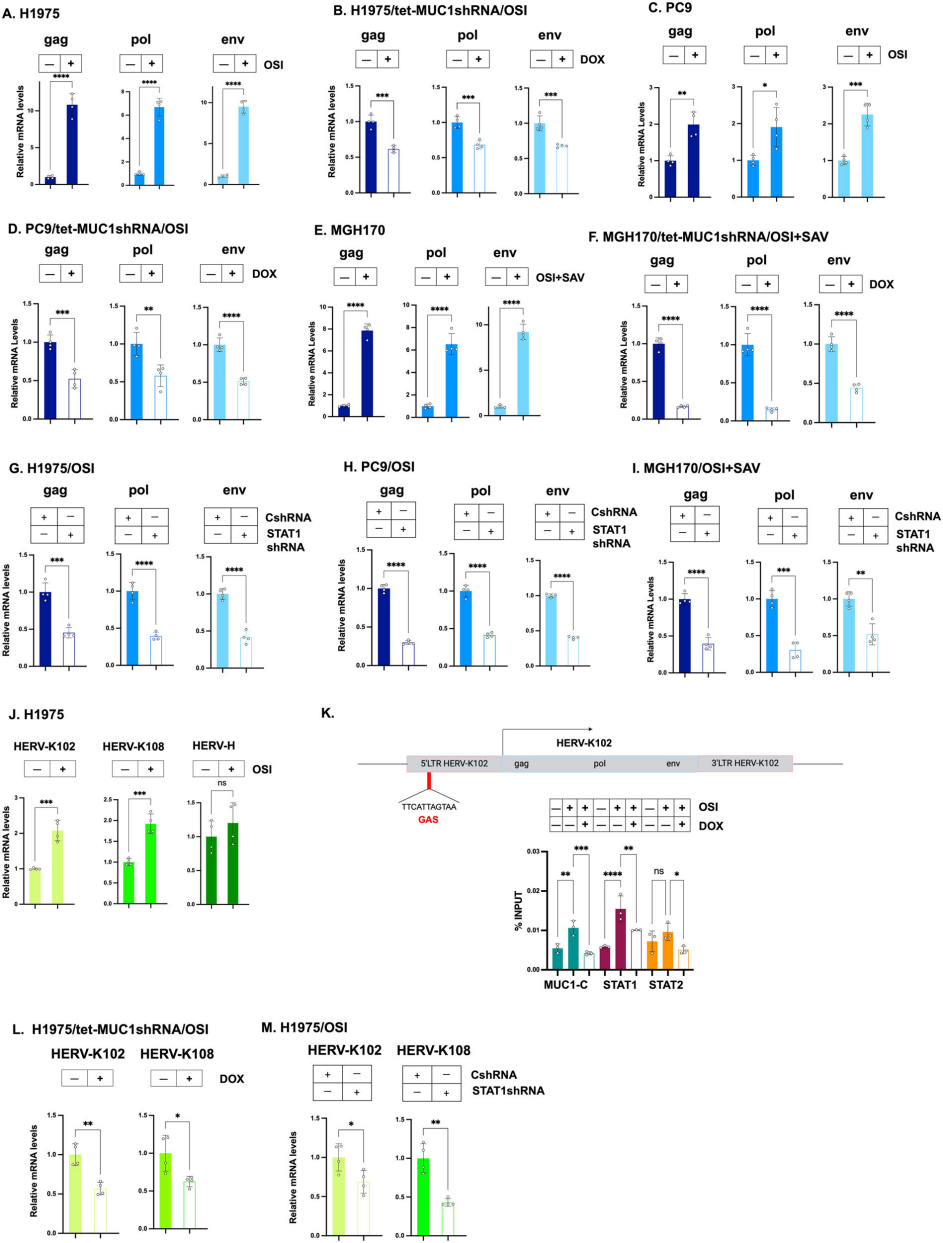
MUC1-C regulates HERV-K expression by a type II IFN pathway. **A** H1975 cells treated with 1 μM OSI for 3 days were analyzed for HERV-K gag, pol and env transcripts by qRT-PCR. The results (mean ± SD of 4 determinations) are expressed as relative levels compared to that obtained for H1975 cells (assigned a value of 1). **B** H1975/tet-MUC1shRNA cells treated with 1 μM OSI for 3 days and vehicle or DOX for 7 days were analyzed for HERV-K gag, pol and env transcripts by qRT-PCR. The results (mean ± SD of 4 determinations) are expressed as relative levels compared to that obtained for vehicle-treated cells (assigned a value of 1). **C** PC9 cells treated with 1 μM OSI for 3 days were analyzed for HERV-K gag, pol and env transcripts by qRT-PCR. The results (mean ± SD of 4 determinations) are expressed as relative levels compared to that obtained for vehicle-treated cells (assigned a value of 1). **D** PC9/tet-MUC1shRNA cells treated with 1 μM OSI for 3 days and vehicle or DOX for 7 days were analyzed for HERV-K gag, pol and env transcripts by qRT-PCR. The results (mean ± SD of 4 determinations) are expressed as relative levels compared to that obtained for vehicle-treated cells (assigned a value of 1). **E** MGH170 cells treated with OSI + SAV for 3 days were analyzed for HERV-K gag, pol and env transcripts by qRT-PCR. The results (mean ± SD of 4 determinations) are expressed as relative levels compared to that obtained for vehicle-treated cells (assigned a value of 1). **F** MGH170/tet-MUC1shRNA cells treated with OSI + SAV for 3 days and vehicle or DOX for 7 days were analyzed for HERV-K gag, pol and env transcripts by qRT-PCR. The results (mean ± SD of 4 determinations) are expressed as relative levels compared to that obtained for vehicle-treated cells (assigned a value of 1). **G** H1975/CshRNA and H1975/STAT1shRNA cells treated with 1 μM OSI for 3 days were analyzed for HERV-K gag, pol and env transcripts by qRT-PCR. The results (mean ± SD of 4 determinations) are expressed as relative levels compared to that obtained for CshRNA cells (assigned a value of 1). **H** PC9/CshRNA and PC9/STAT1shRNA cells treated with 1 μM OSI for 3 days were analyzed for HERV-K gag, pol and env transcripts by qRT-PCR. The results (mean ± SD of 4 determinations) are expressed as relative levels compared to that obtained for CshRNA cells (assigned a value of 1). **I** MGH170/CshRNA and MGH170/STAT1shRNA cells treated with OSI + SAV for 3 days were analyzed for HERV-K gag, pol and env transcripts by qRT-PCR. The results (mean ± SD of 4 determinations) are expressed as relative levels compared to that obtained for CshRNA cells (assigned a value of 1). **J** H1975 cells treated with 1 μM OSI for 3 days were analyzed for the indicated HERV transcripts by qRT-PCR. The results (mean ± SD of 4 determinations) are expressed as relative levels compared to that obtained for H1975 cells (assigned a value of 1). **K** Schema of the HERV-K102 gene with highlighting of a GAS motif in the 5’LTR. Soluble chromatin from H1975 cells treated with 1 μM OSI for 3 days was precipitated with antibodies against MUC1-C, STAT1, STAT2 and IRF9. The DNA samples were amplified by qRT-PCR with primers for the HERV-K102 5’LTR region. The results (mean ± SD of 4 determinations) are expressed as % input. **L** H1975/tet-MUC1shRNA cells treated with 1 μM OSI for 3 days and vehicle or DOX for 7 days were analyzed for HERV-K102 and HERV-K108 transcripts by qRT-PCR. The results (mean ± SD of 4 determinations) are expressed as relative levels compared to that obtained for vehicle-treated cells (assigned a value of 1). **M** H1975/CshRNA and H1975/STAT1shRNA cells treated with 1 μM OSI for 3 days were analyzed for HERV-K102 and HERV-K108 transcripts by qRT-PCR. The results (mean ± SD of 4 determinations) are expressed as relative levels compared to that obtained for CshRNA cells (assigned a value of 1).

*A3* genes evolved in mammals to restrict replication of endogenous retroviruses [1, 2]. More recently integrated and conserved human retroviruses (HERVs) are represented by the HERV-K (HML-2) family [36, 37]. Intriguingly, we found that HERV-K *gag, pol* and *env* gene expression is upregulated in OSI-treated H1975 cells (Fig. 5A). Moreover, as found for A3s, expression of these HERV-K genes was suppressed by targeting MUC1-C genetically (Fig. 5B) and pharmacologically with GO-203 (Fig. S5A). These unanticipated results were confirmed in PC9 (Fig. 5C, D) and MGH170 (Fig. 5E, F) cells. Silencing STAT1 in H1975 cells also suppressed HERV-K *gag, pol* and *env* expression (Fig. 5G); whereas, targeting STAT2 had little if any effect (Fig. S5B). Similar results were obtained in PC9 cells (Figs. 5H and S5C); that is, suppression HERV-K *gag, pol*, and *env* transcripts by STAT1 and not STAT2 silencing. In MGH170 cells, silencing both STAT1 and STAT2 decreased HERV-K *gag, pol*, and *env* expression (Figs. 5I and S5D), which may reflect differences in cross-talk between the STAT1 and STAT2 pathways in H1975, PC9 and MGH170 cells.

In further defining dependencies for HERV-K expression, we found that HERV-K102 and HERV-K108, but not HERV-H, are significantly upregulated in OSI-treated H1975 (Fig. 5J), PC9 (Fig. S5E) and MGH170 cells (Fig. S5F). In addition to type I IFN signaling, MUC1-C promotes activation of the type II IFN pathway (Fig. S5G), which induces the formation of STAT1 homodimer (U-GAF) transcriptional complexes that drive ISGs with GAS elements [30, 31]. The 5’LTRs of HERV-K102 (1q22) and HERV-K108 (3q12.3) include consensus GAS TTCATTAGTAA elements for binding of U-GAF (Figs. 5K and S5H). Analysis of the HERV-K102 5’LTR demonstrated occupancy of MUC1-C and STAT1, but not STAT2 (Fig. 5K), consistent with involvement of U-GAF complexes. By extension, silencing MUC1-C (Fig. 5L) and STAT1 (Fig. 5M), but not STAT2 (Fig. S5I), suppressed HERV-K102 and HERV-K108 expression. STAT1 homodimers activate GAS elements in the absence and presence of IRF1 [32, 33]. Along these lines, silencing IRF1 had little if any effect on (i) HERV-K *gag, pol*, and *env* (Fig. S5J) and (ii) HERV-K102 and HERV-K108 (Fig. S5K) expression. These findings indicate that MUC1-C→type II IFN STAT1 U-GAF→GAS signaling induces HERV-K expression.

### MUC1-C integrates induction of A3 and HERV-K expression by a STING-dependent mechanism

**Fig. 6:**
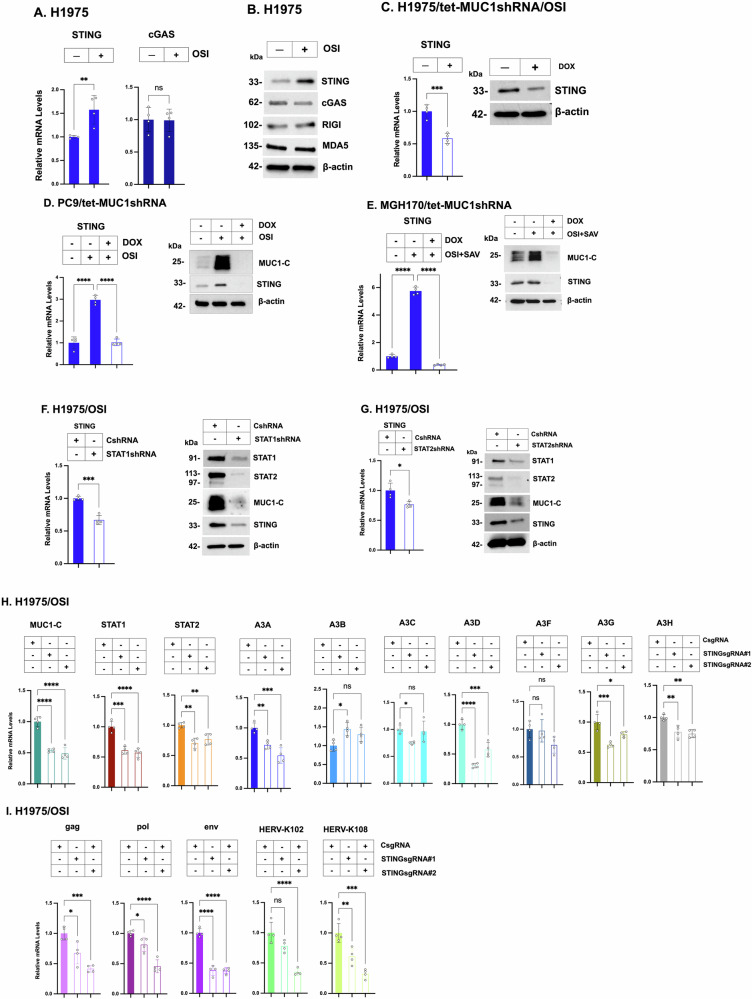
MUC1-C→STING/STAT1 auto-inductive pathway integrates regulation of A3 and HERV expression. **A**, **B** H1975 cells treated with 1 μM OSI for 3 days were analyzed for cGAS and STING transcripts by qRT-PCR. The results (mean ± SD of 4 determinations) are expressed as relative levels compared to that obtained for H1975 cells (assigned a value of 1) (**A**). Lysates were immunoblotted with antibodies against the indicated proteins (**B**). **C** H1975/tet-MUC1shRNA cells treated with 1 μM OSI for 3 days and vehicle or DOX for 7 days were analyzed for STING transcripts by qRT-PCR. The results (mean ± SD of 4 determinations) are expressed as relative levels compared to that obtained for vehicle-treated cells (assigned a value of 1)(left). Lysates were immunoblotted with antibodies against the indicated proteins (right). **D** PC9/tet-MUC1shRNA cells treated with 1 μM OSI for 3 days and vehicle or DOX for 7 days were analyzed for STING transcripts by qRT-PCR. The results (mean ± SD of 4 determinations) are expressed as relative levels compared to that obtained for vehicle-treated cells (assigned a value of 1)(left). Lysates were immunoblotted with antibodies against the indicated proteins (right). **E** MGH170/tet-MUC1shRNA cells treated with OSI + SAV for 3 days and vehicle or DOX for 7 days were analyzed for STING transcripts by qRT-PCR. The results (mean ± SD of 4 determinations) are expressed as relative levels compared to that obtained for control cells (assigned a value of 1)(left). Lysates were immunoblotted with antibodies against the indicated proteins (right). H1975/CshRNA, H1975/STAT1shRNA (**F**) and H1975/STAT2shRNA (**G**) cells treated with 1 μM OSI for 3 days were analyzed for STING transcripts by qRT-PCR. The results (mean ± SD of 4 determinations) are expressed as relative levels compared to that obtained for CshRNA cells (assigned a value of 1)(left). Lysates were immunoblotted with antibodies against the indicated proteins (right). **H**, **I** H1975 cells expressing CsgRNA, STINGsgRNA#1 or STINGsgRNA#2 were treated with 1 μM OSI for 3 days and analyzed for the indicated transcripts by qRT-PCR. The results (mean ± SD of 4 determinations) are expressed as relative levels compared to that obtained for CshRNA cells (assigned a value of 1).

Dysregulation of the A3 enzymes, as well as HERVs, promotes genomic instability [11, 13, 38, 39]. Activation of the pattern recognition receptors (PRRs) cGAS, RIG-I and MDA5 by genomic instability induces the type I and II IFN pathways and downstream ISGs [32, 33, 40–43]. In H1975-OR vs H1975 cells, we found upregulation of (i) STAT1 and STAT2, and (ii) RIG-I, MDA5 and the cGAS-stimulator of IFN genes (STING), but not cGAS (Fig. S6A). In OSI-treated H1975 cells, we also detected upregulation of STING, but not cGAS, transcripts (Fig. 6A) and protein (Fig. 6B); whereas, there was little effect on expression of the RIG-I and MDA5 PRRs (Fig. 6B). We therefore focused on STING and found that STING expression is MUC1-C-dependent in (i) H1975 and PC9 cells treated with OSI (Fig. 6C, D), and (iii) MGH170 cells treated with OSI + SAV (Fig. 6E). We also found that expression of MUC1-C and STING is suppressed by silencing STAT1 and STAT2 in H1975 (Fig. 6F, G), PC9 (Fig. S6B, S6C) and MGH170 (Fig. S6D, S6E) cells. Consistent with MUC1-C-mediated regulation of STAT1 and STAT2, cross-talk among MUC1-C, STING, STAT1 and STAT2 signaling was also notable with silencing STAT1 and STAT2 in these models.

As confirmation of STING involvement, silencing STING in OSI-treated H1975 cells suppressed induction of (i) MUC1-C, STAT1, STAT2, A3A and A3G (Figs. 6H and S6F), and (ii) HERV-K102 and HERV-K108 expression (Fig. 6I). Consistent with these results, studies of PC9 cells silenced for STING (Fig. S6G, S6H) and treated with the STING inhibitor H-151 [44] (Fig. S6I) demonstrating downregulation of (i) MUC1-C, STAT1, STAT2, A3A and A3G, and (ii) HERV-K102 and HERV-K108 expression. These findings indicate that MUC1-C drives a STAT1/STING auto-inductive inflammatory pathway that integrates activation of the IFN type I/II pathways with dysregulation of A3 and HERV gene expression.

## Discussion

**Fig. 7:**
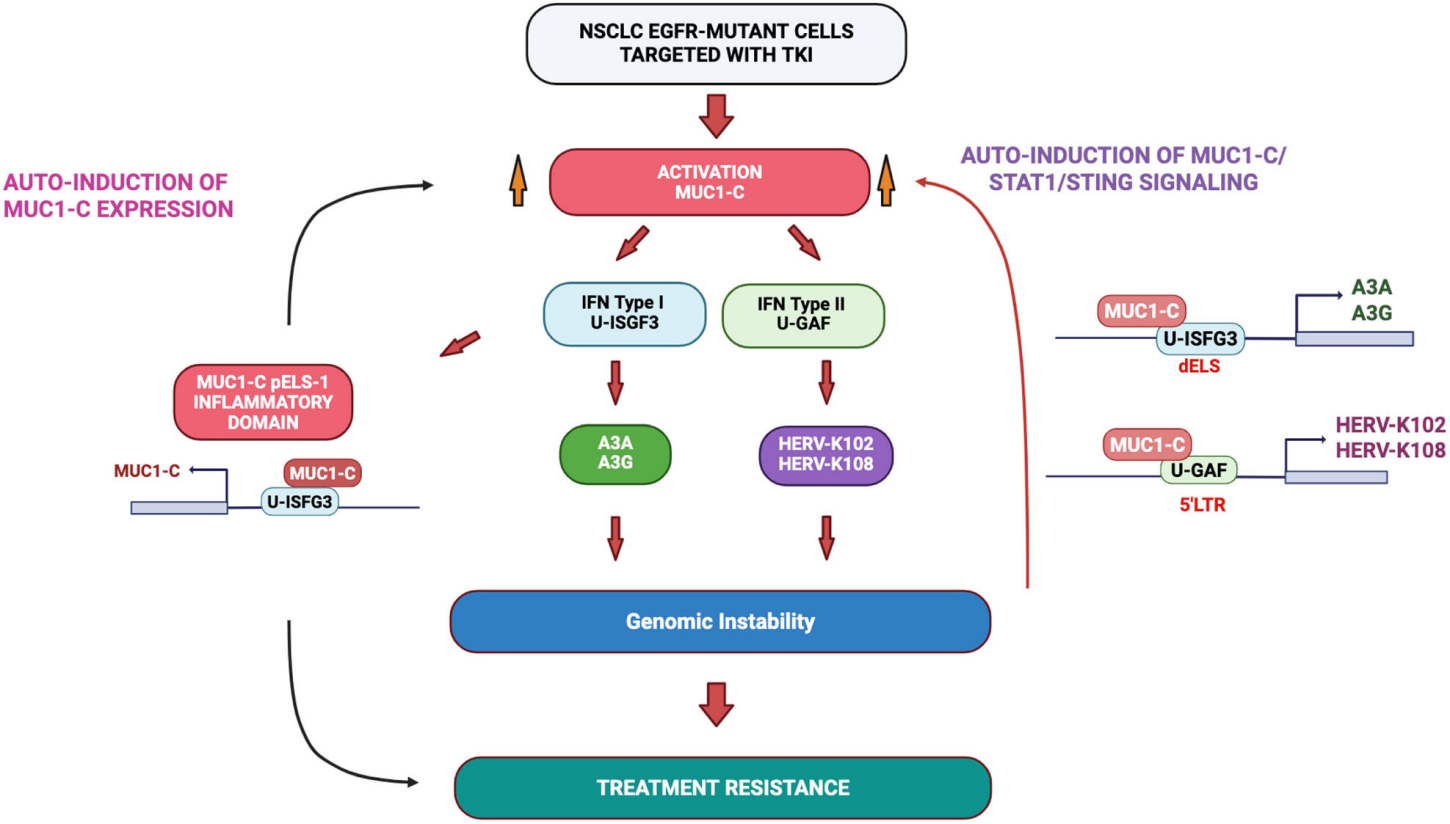
Schema depicting MUC1-C involvement in activating STAT1 signaling and the type I and II IFN pathways in driving APOBEC3 and HERV expression. Treatment of NSCLC EGFR-mutant cells with OSI is associated with induction of MUC1-C expression and OSI resistance [22]. MUC1-C functions as a common effector of the OSI-resistant phenotype linked to EMT, MET amplification and secondary EGFR mutations [22]. The present work extends those findings by demonstrating that MUC1-C drives STAT1 in the response of NSCLC cells to OSI treatment. MUC1-C/STAT1 complexes activate the *MUC1* gene at a pELS region in an auto-inductive inflammatory pathway [23]. Here, we found that the *MUC1* pELS-1 domain is regulated by U-ISGF3 complexes. Our results further demonstrate that the MUC1-C→U-ISGF3 pathway drives ISG A3A and A3G expression. Of significance in this regard, U-ISGF3 also activates ISGs that confer resistance to DNA damage and viruses [61]. Given that MUC1 and A3s co-evolved to protect against viruses, one notion was that MUC1-C→STAT1 signaling could link activation of A3s with induction of HERVs. Indeed, we found that MUC1-C induces HERV-K102/108 by activation of the IFN type II pathway and the formation of U-GAF complexes. These findings supported a model in which MUC1-C integrates induction of A3s and HERVs by a common STAT1-mediated mechanism. Dysregulation of A3 and HERV-K expression has been linked to genomic instability. Along these lines, our results demonstrate that MUC1-C integrates activation of A3 and HERV-K by a shared STING/STAT1-dependent auto-inductive pathway that has the potential for driving A3-induced mutagenesis and treatment resistance. A common denominator among these highly interrelated pathways is that MUC1-C/U-ISGF3 IFN type I complexes integrate activation of the *MUC1* pELS-1 inflammatory domain and A3 expression, which could conceivably be in response to MUC1-C/STAT1 IFN type II-mediated induction of HERV-Ks.

Cancer cells that survive drug treatment and acquire resistance mechanisms promote disease relapse and progression [11, 12, 45]. A3A and A3B are upregulated in NSCLC cells treated with TKIs and confer treatment resistance [13–15]; however, the mechanism underlying their induction has been unclear. We therefore leveraged NSCLC cells as a model to define the regulation of A3s. The present work sheds light on A3 activation by identifying a MUC1-C-dependent pathway that is necessary for expression of A3A, A3G and other members of the A3 family (Fig. 7). We show this previously unrecognized regulation of A3s is conferred by induction of an inflammatory MUC1-C→type I IFN U-ISGF3 pathway that is also responsible for activating the *MUC1* pELS-1 inflammatory domain (Fig. 7). Specifically, for the *A3A* and *A3G* genes, we found that MUC1-C/U-ISGF3 complexes occupy their respective ISRE regions, which are dependent on MUC1-C for STAT1/STAT2/IRF9 occupancy and increases in chromatin accessibility. Silencing MUC1-C, STAT1 and STAT2 also suppressed expression of other *A3* genes in potential support of a common MUC1-C-driven pathway of A3 regulation. MUC1-C is activated by replicative and other forms of stress that, if prolonged as in settings of chronic inflammation, establish irreversible epigenetic changes and cancer [20, 21]. Cancer cells exhibit genomic instability induced by DNA replicative stress that contributes to drug resistance [11]. As a result, MUC1-C-driven activation of *A3* genes, which have been linked to widespread mutations throughout the genomes of cancer cells [46], could contribute to MUC1-C-mediated drug resistance [20–22] (Fig. 7).

MUC1-C functions as a common effector of OSI resistance conferred by induction of EMT, MET amplification and the secondary EGFR(T790M/C797S) mutation [22]. How MUC1-C contributes to these pleotropic mechanisms of OSI resistance was not addressed in those studies. Nonetheless, other work uncovered that MUC1-C is activated in a STAT1 auto-inductive pathway that drives drug resistance [24]. Inflammatory memory, also known as trained memory, endows cancer cells with the capacity to acquire resistance to drugs with diverse structures and mechanisms of action [47]. Along these lines, the *MUC1* pELS-1 region represents a potential inflammatory memory domain that recalls OSI exposure to more effectively respond to subsequent TKI treatment [22]. The present work uncovered involvement of U-ISGF3 in regulation of the *MUC1* pELS-1, which is of significance in that U-ISGF3 was also identified in driving A3A and A3G. These findings supported activation of MUC1 and A3s by a common U-ISGF3-dependent inflammatory pathway (Fig. 7).

Stress is linked to dysregulation of HERV expression [48]. The present results reveal the unanticipated finding that MUC1-C is necessary for activation of the HERV-K *gag, pol* and *env* genes (Fig. 7). The HERV-K family subgroup HML-2 represents the most recently integrated proviruses that are biologically active in association with promoting chronic inflammation [36, 37, 49, 50]. Here, we found that, in contrast to the A3s, MUC1-C→STAT1 U-GAF signaling activates the HERV-K102 and HERV-K108 genes. MUC1-C/STAT1 complexes occupy the HERV-K102 5’LTR, which includes a GAS element linked to driving HERV-K102 in inflammatory and neurodegenerative disorders [49–51]. The findings that MUC1-C and STAT1 are necessary for activation of HERV-K102 and HERV-K108, as well as HERV-K *gag, pol* and *env* genes, supported involvement of the MUC1-C→type II IFN STAT1 U-GAF pathway. Selectivity for HERV-K was further supported by lack of an effect on HERV-H expression. A question in need of further investigation is whether induction of HERV-Ks by the IFN type II pathway and thereby viral mimicry contributes to U-ISGF3-mediated activation of the *MUC1* pELS-1 inflammatory domain and A3 expression (Fig. 7).

*MUC1* appeared in mammals to protect respiratory tract epithelia and other barrier tissues from loss of homeostasis by viral infections [18] The A3 enzymes evolved in mammals to protect against replication of ERVs [2, 10]. The co-evolution of MUC1 and A3s is intriguing in that many of the genes which appeared in mammals function in promoting placentation and survival of offspring. Whether MUC1 and A3s co-evolved to protect against exogenous and endogenous retroviruses will require additional study. Nonetheless, our findings support a role for MUC1-C in integrating the regulation of A3s and HERVs (Fig. 7). MUC1-C activates inflammatory signaling pathways in the wound healing response [20, 21, 52]. In cancer cells, MUC1-C drives auto-inductive chronic inflammatory signaling involving STAT1-mediated regulation of STING and downstream ISGs that amplify the type I and II IFN pathways [23–26]. The present results demonstrate that a (i) MUC1-C→STING/type I IFN/U-ISGF3 pathway drives expression of A3s, and (ii) MUC1-C→STING/type II IFN U-GAF pathway drives HERV-K genes (Fig. 7). Common MUC1-C→STING/STAT1 signaling uncovered here in integrating A3 and HERV expression is likely a response which, in principle, is reversible with reestablishment of homeostasis. However, chronic activation of MUC1-C→STING/STAT1 signaling in NSCLC and other cancer cells could constitute an irrevocable response that drives genomic instability and drug-resistant cancer progression (Fig. 7).

Treatment of NSCLCs with targeted and immunotherapeutic agents has been associated with markedly diverse mechanisms of resistance that include (i) transformation to other histological subtypes [53], (ii) suppression of ferroptosis [54], and (iii) alterations in the tumor microenvironment [55], among others [56]. Dysregulation of A3s contributes to treatment resistance and immune escape across pan-cancers [57, 58]. A provocative aspect of the present work is that the MUC1-C-induced MUC1-C→U-ISGF3 inflammatory response is linked to induction of A3s as an adaptation to viral infections, but could also represent a maladaptation to treatment of cancer cells with agents that contribute to viral mimicry and auto-activation of the IFN type I pathway [59].

## Methods

### All methods were performed in accordance with the relevant guidelines and regulations

#### Cell culture

H1975/EGFR(L858R/T790M) (ATCC) and PC9/EGFR(19del) cells (Millipore Sigma, Burlington, MA, USA) were cultured in RPMI1640 medium (Thermo Fisher Scientific, Waltham, MA, USA) supplemented with 10% fetal bovine serum (FBS; GEMINI Bio-Products, West Sacramento, CA, USA). MGH170-1D #2 (*MET* amplification) cells [35] were cultured in RPMI1640 medium supplemented with 10% FBS and 5% glutamine. Cells were treated with GO-203, 1 μM osimertinib (OSI) and 1 μM savolitinib (SAV) (Selleck Chemicals, Houston, TX, USA). Authentication of the cells was performed every 3–4 months by short tandem repeat (STR) analysis. Cells were monitored for mycoplasma contamination every 3–4 months using the MycoAlert Mycoplasma Detection Kit (Lonza, Rockland, MA, USA). Cells were maintained for 3 months when performing experiments.

#### Gene silencing

MUC1shRNA (MISSION shRNA TRCN0000122938; Sigma, St. Louis, MO, USA) or a control scrambled shRNA (CshRNA; Sigma) was inserted into the pLKO-tet-puro vector (Plasmid #21915; Addgene, Cambridge, MA, USA) as described [22]. The MUC1shRNA#2 (MISSION shRNA TRCN0000430218), STAT1shRNA (MISSION shRNA TRCN0000004266), STAT2shRNA (MISSION shRNA TRCN0000364400), IRF1shRNA#1 (MISSION shRNA TRCN0000014672), IRF1shRNA#2 (MISSION shRNA TRCN0000218951) and STINGsgRNAs [60] were produced in HEK293T cells as described [22]. Flag-tagged MUC1-CD was inserted into the empty control pLenti CMV Blast DEST(706-1) vector (Plasmid #17451; Addgene) as described [22]. Vector-transduced cells were selected for growth in 1–2 μg/ml puromycin. Cells were treated with 0.1% DMSO as the vehicle control or 500 ng/ml doxycycline (DOX; Millipore Sigma).

#### Quantitative reverse-transcription PCR (qRT-PCR)

Total cellular RNA was isolated using Trizol reagent (Thermo Fisher Scientific). cDNAs were synthesized using the High Capacity cDNA Reverse Transcription Kit (Applied Biosystems, Grand Island, NY, USA) as described [22]. The cDNA samples were amplified using the Power SYBR Green PCR Master Mix (Applied Biosystems) and the CFX96 Real-Time PCR System (BIO-RAD, Hercules, CA, USA) as described [22]. Primers used for qRT-PCR are listed in Supplementary Table 1.

#### Analysis of A3A activity

A3A RNA editing was analyzed by digital PCR for UpC sites within the DDOST gene mRNA (DDOST 558C→U) as described [13].

#### Immunoblot analysis

Total lysates prepared from non-confluent cells were subjected to immunoblot analysis using anti-MUC1-C (HM-1630-P1ABX, 1:1000 dilution; Thermo Fisher Scientific, Waltham, MA, USA), anti-β-actin (A5441, 1:5000 dilution; Sigma-Aldrich, Burlington, MA, USA), anti-pSTAT1 (#9177S, 1:1000; Cell Signaling Technology (CST), Danvers, MA, USA), anti-STAT1 (#9172, 1:1000; CST); anti-pSTAT2 (#88410S, 1:1000; CST), anti-STAT2 (#72604, 1:1000; CST), anti-IRF9 (#76684, 1:1000; CST), anti-A3C (), anti A3F (), anti-A3G (), anti-IRF1 (#8478, 1:1000; CST), anti-cGAS (#15102, 1:1000; CST), anti-STING (#13647, 1:1000; CST), anti-MDA5 (#5321, 1:1000; CST) and anti-RIG-I (#3743, 1:1000; CST) as described [22, 32, 33].

#### Chromatin immunoprecipitation

Chromatin immunoprecipitation (ChIP) was performed using control IgG (Santa Cruz Biotechnology), anti–MUC1-C (#MA5–11202, Thermo Fisher Scientific), anti-STAT1 (#ab109320, Abcam), anti-STAT2 (#8478, CST), anti-IRF9 (#76684, CST) and anti-IRF1 (#8478; CST) as described [61]. Precipitated DNAs were detected by PCR using the primers listed in Supplementary Table S2. The immunoprecipitated DNA was quantified using SYBR-green and the CFX96 Touch Real-Time PCR Detection System (Bio-Rad) as described [60]. Data are reported as percentage of input DNA as described [60].

#### Chromatin accessibility assays

DNAse1 chromatin accessibility assays were performed on chromatin isolated as described [61]. Aliquots of chromatin were left untreated or digested with 3 U/100 μl DNase I (Promega, Madison, WI, USA) for 4 min at room temperature. DNA was purified and amplified by qPCR using primers listed in Supplementary Table S2. qPCR results were analyzed according to the formula 100/2Ct (DNase I) −Ct (no DNase I). The data were normalized to input DNA without DNase I treatment as described [60].

#### Statistical analysis

Each experiment was performed at least three times. Unpaired two-tailed Student’s *t*-tests were used to assess differences between the mean ± SD of two groups. *P*-values were considered significant at *p* < 0.05. GraphPad Prism9 was used for all statistical analyses. Asterisks represent *P ≤ 0.05, **P ≤ 0.01, ***P ≤ 0.001, ****P ≤ 0.0001 with CI = 95%.

## Supplementary information

Supplemental Information

Western Blots source data

## Data Availability

All data supporting the findings in this study are available within the article and the Supplementary information files. The accession numbers for the RNA-seq data are GEO Submissions GSE263757, GSE270995 and GSE270997.
