# Supplementary material for: Activation of APOBEC3 cytidine deaminases and endogenous retroviruses is integrated by MUC1-C in NSCLC cells
Source: Cell Death Discov. 2025 Aug 8;11:372. doi: 10.1038/s41420-025-02673-9 (PMC12334734; doi:10.1038/s41420-025-02673-9)
Supplement: Supplementary file 1 — Supplemental Information [file 41420_2025_2673_MOESM1_ESM.docx]

**Supplemental Figures and Figures Legends**

**Supplemental Figure S1. Effects of targeting MUC1-C in OSI-treated H1975 and PC9 cells. A.** H1975 cells treated with vehicle or 1 μM OSI for the indicated days were analyzed for the indicated transcripts by qRT-PCR. The results (mean±SD of 4 determinations) are expressed as relative levels compared to that obtained for vehicle-treated cells (assigned a value of 1). **B.** Lysates from H1975/tet-MUC1shRNA cells treated with 1 μM OSI for 3 days and vehicle or DOX for 7 days were immunoblotted with antibodies against the indicated proteins. **C.** Lysates from H1975/CshRNA and H1975/MUC1shRNA#2 cells treated with 1 μM OSI for 3 days were immunoblotted with antibodies against the indicated proteins. **D.** H1975/CshRNA and H1975/MUC1shRNA#2 cells treated with 1 μM OSI for 3 days were analyzed for the indicated transcripts by qRT-PCR. The results (mean±SD of 4 determinations) are expressed as relative levels compared to that obtained for vehicle-treated DTPs (assigned a value of 1). **E.** PC9 cells treated with vehicle or 1 μM OSI for the indicated days were analyzed for the indicated transcripts by qRT-PCR. The results (mean±SD of 4 determinations) are expressed as relative levels compared to that obtained for vehicle-treated cells (assigned a value of 1). **F.** Lysates from PC9/tet-MUC1shRNA cells treated with 1 μM OSI for 3 days and vehicle or DOX for 7 days were immunoblotted with antibodies against the indicated proteins.

**Supplemental Figure S2. Regulation of A3A by a MUC1-C**→**U-ISGF3 pathway. A.** GSEA of RNA-seq data from biological triplicates of H1975/tet-MUC1shRNA cells treated with vehicle or DOX for 7 days using the HALLMARK INTERFERON ALPHA RESPONSE gene signature. **B and C.** H1975 (**B**) and PC9 (**C**) cells treated with vehicle or 1 μM OSI for the indicated days were analyzed for the indicated transcripts by qRT-PCR using primers. The results (mean±SD of 4 determinations) are expressed as relative levels compared to that obtained for vehicle-treated cells (assigned a value of 1). **D and E.** Chromatin from H1975 (**D**) and PC9 (**E**) cells treated with 1 μM OSI for 3 days was analyzed for accessibility of the *MUC1* pELS-1 region by nuclease digestion. The results (mean±SD of 4 determinations) are expressed as % undigested chromatin. **F.** Genome browser snapshot of ATAC-seq data from the *MUC1* pELS-1 region in PC9 cells treated with vehicle or 1 μM OSI for 3 days. **G and H.** H1975 (**G**) and PC9 (**H**) cells treated with 1 μM OSI and vehicle or 1 μM GO-203 for 3 days were analyzed for the indicated transcripts by qRT-PCR. The results (mean±SD of 4 determinations) are expressed as relative levels compared to that obtained for vehicle-treated cells (assigned a value of 1). **I and J.** PC9/CshRNA and PC9/STAT1shRNA (**I**) or PC9/STAT2shRNA (**J**) cells treated with 1 μM OSI for 3 days were analyzed for the indicated transcripts by qRT-PCR. The results (mean±SD of 4 determinations) are expressed as relative levels compared to that obtained for CshRNA cells (assigned a value of 1).

**Supplemental Figure S3. MUC1-C regulates A3A in MGH170 cells. A.** MGH170 cells treated with OSI+SAV and vehicle or 1 μM GO-203 for 3 days were analyzed for the indicated transcripts by qRT-PCR. The results (mean±SD of 4 determinations) are expressed as relative levels compared to that obtained for vehicle-treated cells (assigned a value of 1). **B**-**D**. Kaplan-Meier curves for overall survival (OS) of NSCLC patients according to high (red) or low (black) expression of the A3A, A3B (**B**), A3C, A3D, A3G, A3H (**C**), and A3F (**D**) genes using the plotter data base (54). The cutoff value for each A3 gene was set at its respective median expression level. p-values were obtained using the log rank test.

**
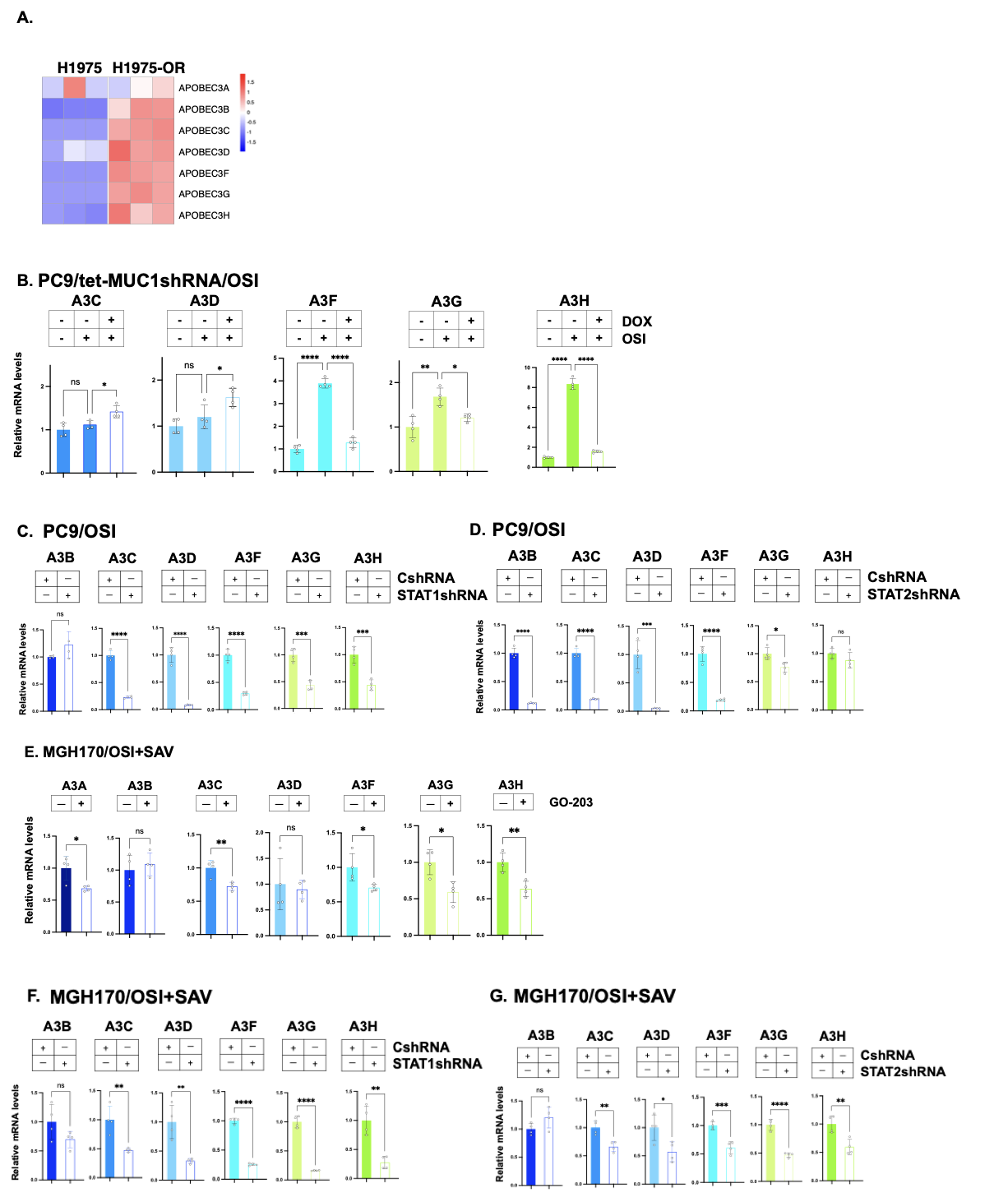
**

**Supplemental Figure S4. Silencing STAT1 and STAT2 suppresses expression of A3s**. **A.** Heatmap of A3 expression in H1975 vs H1975-OR cells**. B.** PC9/tet-MUC1shRNA cells treated with 1 μM OSI for 3 days and vehicle or DOX for 7 days were analyzed for the indicated transcripts by qRT-PCR. The results (mean±SD of 4 determinations) are expressed as relative levels compared to that obtained for control cells (assigned a value of 1). **C and D.** PC9/CshRNA, PC9/STAT1shRNA (**C**) and PC9/STAT2shRNA (**D**) cells treated with 1 μM OSI for 3 days were analyzed for the indicated transcripts by qRT-PCR. The results (mean±SD of 4 determinations) are expressed as relative levels compared to that obtained for CshRNA cells (assigned a value of 1). **E.** MGH170 cells treated with OSI+SAV and vehicle or 1 μM GO-203 for 2 days were analyzed for the indicated transcripts by qRT-PCR. The results (mean±SD of 4 determinations) are expressed as relative levels compared to that obtained for vehicle-treated cells (assigned a value of 1). **F and G.** MGH170/CshRNA, MGH170/STAT1shRNA (**F**) and MGH170/STAT2shRNA (**G**) cells treated with OSI+SAV were analyzed for the indicated transcripts by qRT-PCR. The results (mean±SD of 4 determinations) are expressed as relative levels compared to that obtained for CshRNA cells (assigned a value of 1).

**Supplemental Figure S5. MUC1-C regulates HERV expression in NSCLC cells. A.** H1975 cells treated with 1 μM OSI and vehicle or 1 μM GO-203 for 3 days were analyzed for HERV-K gag, pol and env transcripts by qRT-PCR. The results (mean±SD of 4 determinations) are expressed as relative levels compared to that obtained for vehicle-treated cells (assigned a value of 1). **B.** H1975/CshRNA and H1975/STAT2shRNA cells treated with 1 μM OSI for 3 days were analyzed for the indicated HERV-K transcripts by qRT-PCR. The results (mean±SD of 4 determinations) are expressed as relative levels compared to that obtained for CshRNA cells (assigned a value of 1). **C.** PC9/CshRNA and PC9/STAT2shRNA cells treated with 1 μM OSI for 3 days were analyzed for the indicated HERV-K transcripts by qRT-PCR. The results (mean±SD of 4 determinations) are expressed as relative levels compared to that obtained for CshRNA cells (assigned a value of 1). **D.** MGH170/CshRNA and MGH170/STAT2shRNA treated with OSI+SAV for 3 days were analyzed for the indicated HERV-K transcripts by qRT-PCR. The results (mean±SD of 4 determinations) are expressed as relative levels compared to that obtained for CshRNA cells (assigned a value of 1). **E and F.** PC9 cells treated with 1 μM OSI for 3 days (**E**) and MGH170 cells treated with OSI+SAV for 3 days (**F**) were analyzed for the indicated HERV transcripts by qRT-PCR. The results (mean±SD of 4 determinations) are expressed as relative levels compared to that obtained for control PC9 and MGH170 cells (assigned a value of 1). **G.** GSEA of RNA-seq data from biological triplicates of H1975/tet-MUC1shRNA cells treated with vehicle or DOX for 7 days using the HALLMARK INTERFERON GAMMA RESPONSE gene signature. **H.** Schema of the HERV-K108 gene with highlighting of a GAS motif in the 5’LTR. **I.** H1975/CshRNA and H1975/STAT2shRNA cells treated with 1 μM OSI for 3 days were analyzed for HERV-K102 and HERV-K108 transcripts by qRT-PCR. The results (mean±SD of 4 determinations) are expressed as relative levels compared to that obtained for CshRNA cells (assigned a value of 1). **J and K.** H1975/CshRNA, H1975/IRF1shRNA#1 (**J**) and H1975/IRF1shRNA#2 (**K**) cells treated with 1 μM OSI for 3 days were analyzed for the indicated transcripts by qRT-PCR. The results (mean±SD of 4 determinations) are expressed as relative levels compared to that obtained for CshRNA cells (assigned a value of 1).

**Supplemental Figure S6. MUC1-C signaling integrates regulation of A3 and HERV expression. A.** Heatmap depicting expression of the indicated genes in H1975 and H1975-OR cells. **B and C.** PC9/CshRNA, PC9/STAT1shRNA (**B**) and PC9/STAT2shRNA (**C**) cells treated with 1 μM OSI for 3 days were analyzed for STING transcripts by qRT-PCR. The results (mean±SD of 4 determinations) are expressed as relative levels compared to that obtained for CshRNA cells (assigned a value of 1)(left). Lysates were immunoblotted with antibodies against the indicated proteins (right). **D and E.** MGH170/CshRNA, MGH170/STAT1shRNA (**D**) and MGH170/STAT2shRNA (**E**) cells treated with OSI+SAV for 3 days were analyzed for STING transcripts by qRT-PCR. The results (mean±SD of 4 determinations) are expressed as relative levels compared to that obtained for CshRNA cells (assigned a value of 1)(left). Lysates were immunoblotted with antibodies against the indicated proteins (right). **F and G.** Lysates from H1975 (**F**) and PC9 (**G**) cells expressing CsgRNA, STINGsgRNA#1 or STINGsgRNA#2 were treated with 1 μM OSI for 3 days and immunoblotted with antibodies against the indicated proteins. **H.** PC9 cells expressing CsgRNA, STINGsgRNA#1 or STINGsgRNA#2 were treated with 1 μM OSI for 3 days and analyzed for the indicated transcripts by qRT-PCR. The results (mean±SD of 4 determinations) are expressed as relative levels compared to that obtained for CshRNA cells (assigned a value of 1). **I.** PC9 cells treated with vehicle or 1 μM H-151 for 4 days before adding 1 μM OSI for an additional 3 days were analyzed for the indicated transcripts by qRT-PCR. The results (mean±SD of 4 determinations) are expressed as relative levels compared to that obtained for vehicle-treated cells (assigned a value of 1).

**Supplemental Tables**

**Supplemental Table S1. Primers used for qRT-PCR analysis.**

| MUC1-C | FWD | TACCGATCGTAGCCCCTATG |
| --- | --- | --- |
|  | REV | CTCACCAGCCCAAACAGG |
| STAT1 | FWD | GGAACTTGATGGCCCTAAAGGA |
|  | REV | ACAGAGCCCACTATCCGAGACA |
| STAT2 | FWD | GCAGCACAATTTGCGGAA |
|  | REV | ACAGGTGTTTCGAGAACTGGC |
| IRF9 | FWD | CCCGACCTCACCGATGAC |
|  | REV | TCTCGCGAAGCTGGATGTC |
| APOBEC3A | FWD | GAGAAGGGACAAGCACATGG |
|  | REV | TGGATCCATCAAGTGTCTGG |
| APOBEC3B | FWD | GACCCTTTGGTCCTTCGAC |
|  | REV | GCACAGCCCCAGGAGAAG |
| APOBEC3C | FWD | AGCGCTTCAGAAAAGAGTGG |
|  | REV | AAGTTTCGTTCCGATCGTTG |
| APOBEC3D | FWD | ACCCAAACGTCAGTCGAATC |
|  | REV | CACATTTCTGCGTGGTTCTC |
| APOBEC3F | FWD | CCGTTTGGACGCAAAGAT |
|  | REV | CCAGGTGATCTGGAAACACTT |
| APOBEC3G | FWD | CCGAGGACCCGAAGGTTAC |
|  | REV | TCCAACAGTGCTGAAATTCG |
| APOBEC3H | FWD | AGCTGTGGCCAGAAGCAC |
|  | REV | CGGAATGTTTCGGCTGTT |
| HERV-K 102 | FWD | AGAAAAGGGCCTCCACGGAGATG |
|  | REV | ATCCTGGTGCTCTCCCTAGG |
| HERV-K 108 | FWD | GTATGCTGCTTGCAGCCTTGATGAT |
|  | REV | GTGACATCCCGCTTACCATG |
| HERV-K env | FWD | CTGAGGCAATTGCAGGAGTT |
|  | REV | GCTGTCTCTTCGGAGCTGTT |
| HERV-K gag | FWD | AGCAGGTCAGGTGCCTGTAACATT |
|  | REV | TGGTGCCGTAGGATTAAGTCTCCT |
| HERV pol | FWD | TGATCCCMAAAGAYTGGCCTT |
|  | REV | TTAAGCATTCCCTGAGGYAACA |
| HERV-H env | FWD | CTGCAAGCTGGAAGGTTGTG |
|  | REV | AGGGTGACCTGAGGTTGTTG |
| IRF1 | FWD | CATGGCTGGGACATCAACAA |
|  | REV | TTGTATCGGCCTGTGTGAATG |
| STING | FWD | CCAGAGCACACTCTCCGGTA |
|  | REV | CGCATTTGGGAGGGAGTAGTA |
| cGAS | FWD | TAACCCTGGCTTTGGAATCAAAA |
|  | REV | TGGGTACAAGGTAAAATGGCTTT |
| MDA5 | FWD | CCATGGAGAAGGCTGGGG |
|  | REV | CAAAGTTGTCATGGATGACC |
| RIG-I | FWD | CTGGACCCTACCTACATCCTG |
|  | REV | GGCATCCAAAAAGCCACGG |
| β-actin | FWD | GATGAGATTGGCATGGCTTT |
|  | REV | CACCTTCACCGTTCCAGTTT |

**Supplemental Table S2. Primers used for ChIP-qPCR.**

| MUC1-C pELS | FWD | GCTGGAGAACAAACGGGTAG |
| --- | --- | --- |
|  | REV | GAGCAGGTGACAGGTGACAA |
| APOBEC3A dELS | FWD | AAACAGGCCCAACTCCTTCT |
|  | REV | GGGAAGGTTCCTTTCTGTCC |
| APOBEC3G pELS | FWD | CCCCAGAGAAAACCAGAAGAG |
|  | REV | TCCCTAAAGTGACCTCCCAG |
| HERV-K 5’LTR | FWD | CGTGACATCCATTTGCCATAATG |
|  | REV | AAATCTTACACCTGCCCACTC |
