# Supplementary material for: Activation of APOBEC3 cytidine deaminases and endogenous retroviruses is integrated by MUC1-C in NSCLC cells
Source: Cell Death Discov. 2025 Aug 8;11:372. doi: 10.1038/s41420-025-02673-9 (PMC12334734; doi:10.1038/s41420-025-02673-9)
Supplement: Supplementary file 2 — Western Blots source data [file 41420_2025_2673_MOESM2_ESM.pdf]

Figure S1

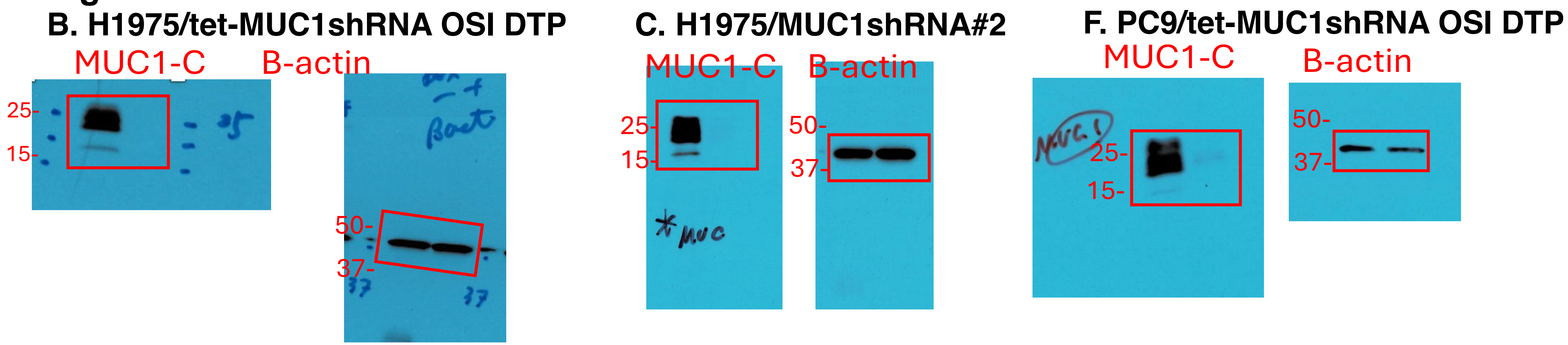

Figure 2

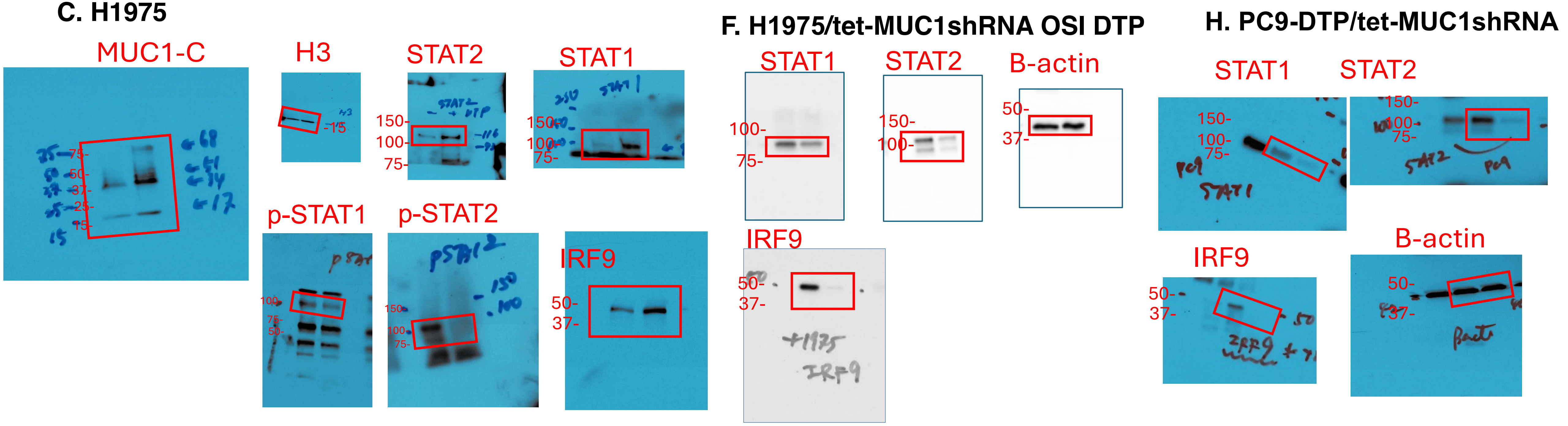

Figure 3

3C

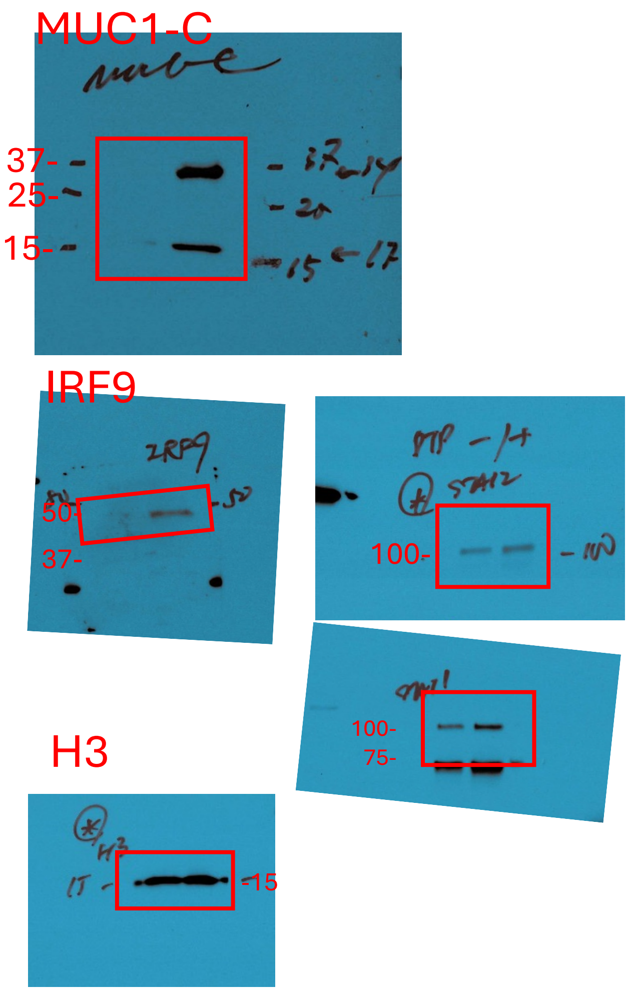

3E

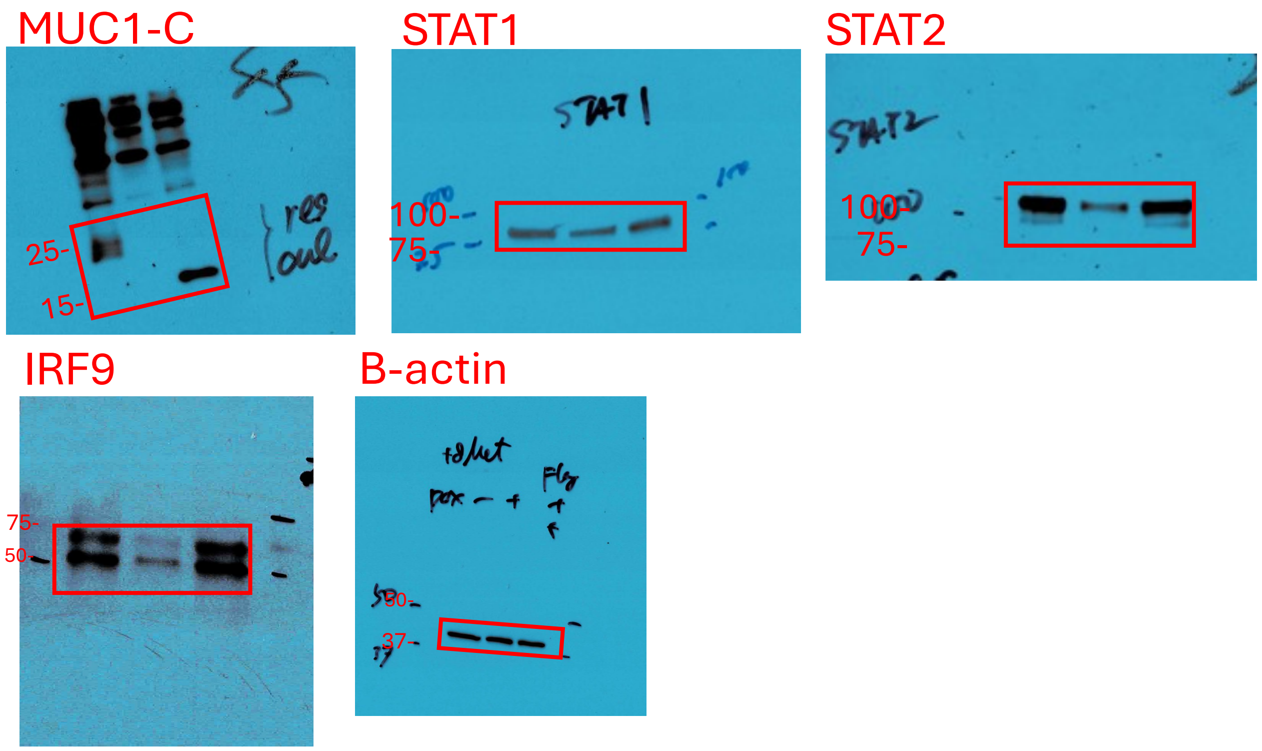

Figure 4

Figure 4F

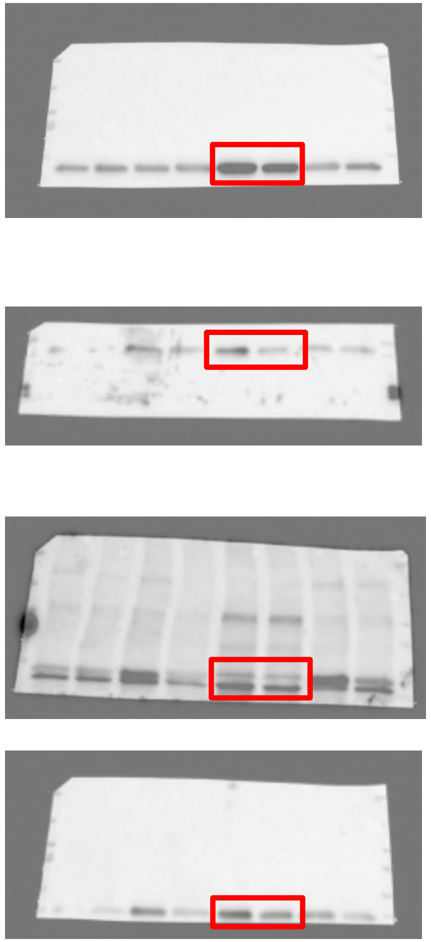

Figure 4K

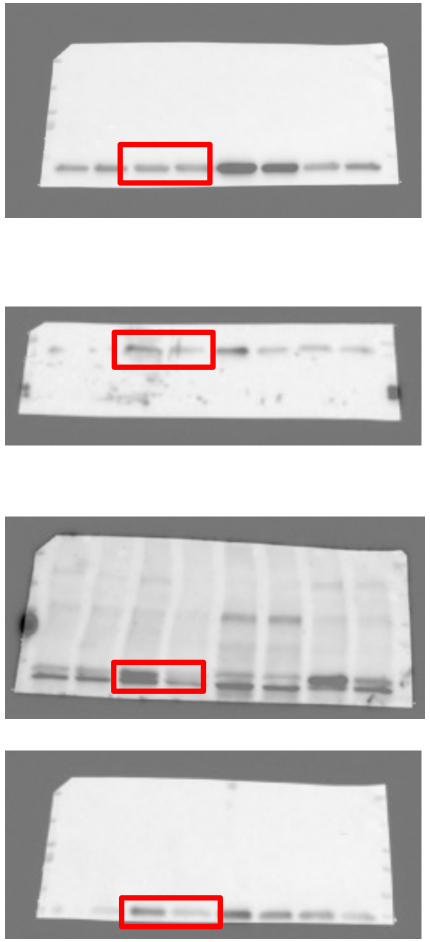

Figure 6

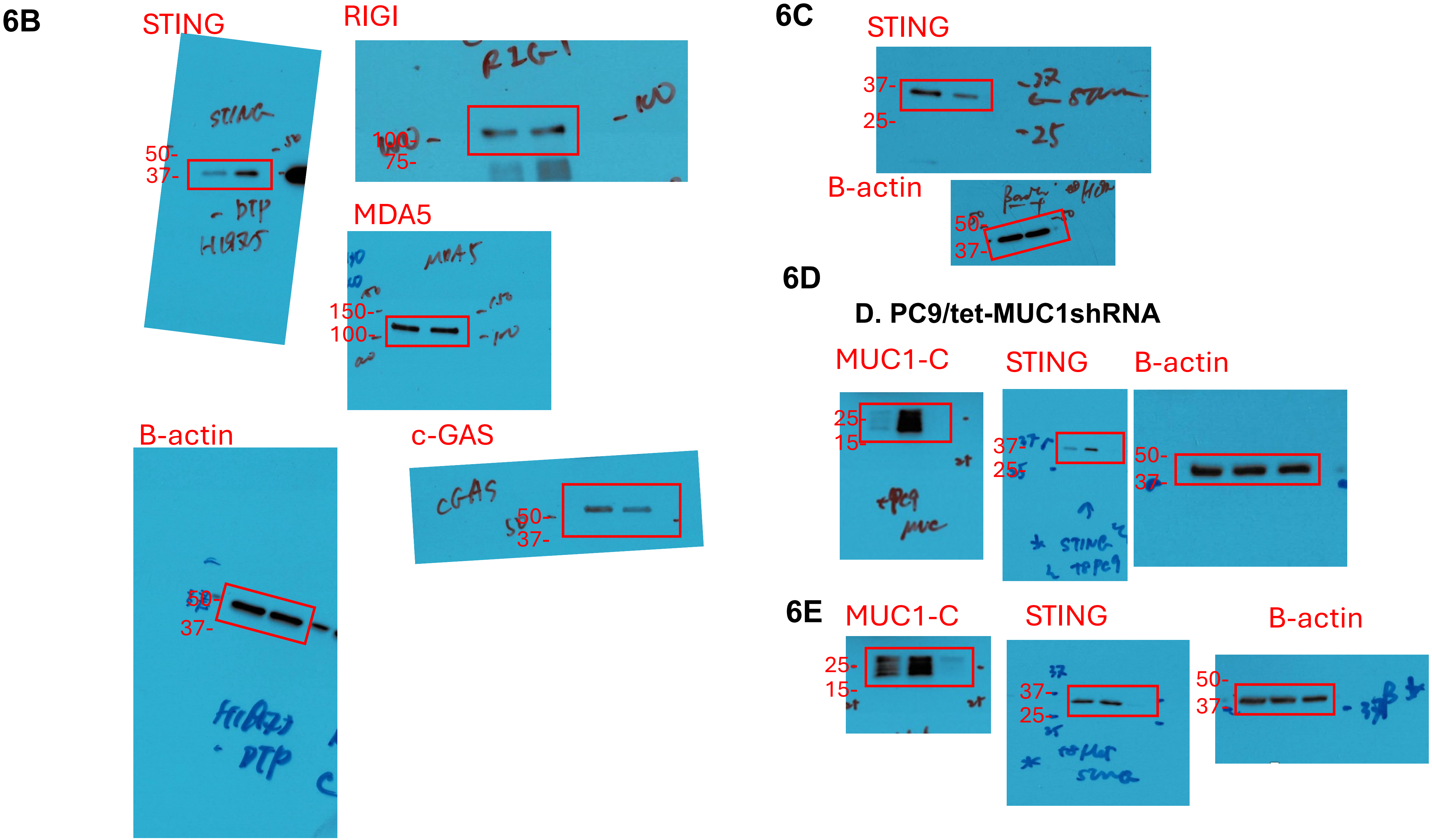
6B
STING
50-
37-
RIGI
100-
75-
MDA5
150-
100-
B-actin
50-
37-
c-GAS
50-
37-
6C
STING
37-
25-
B-actin
50-
37-
6D
D. PC9/tet-MUC1shRNA
MUC1-C
25-
15-
STING
37-
25-
B-actin
50-
37-
6E
MUC1-C
25-
15-
STING
37-
25-
B-actin
50-
37-

Figure 6

終わり

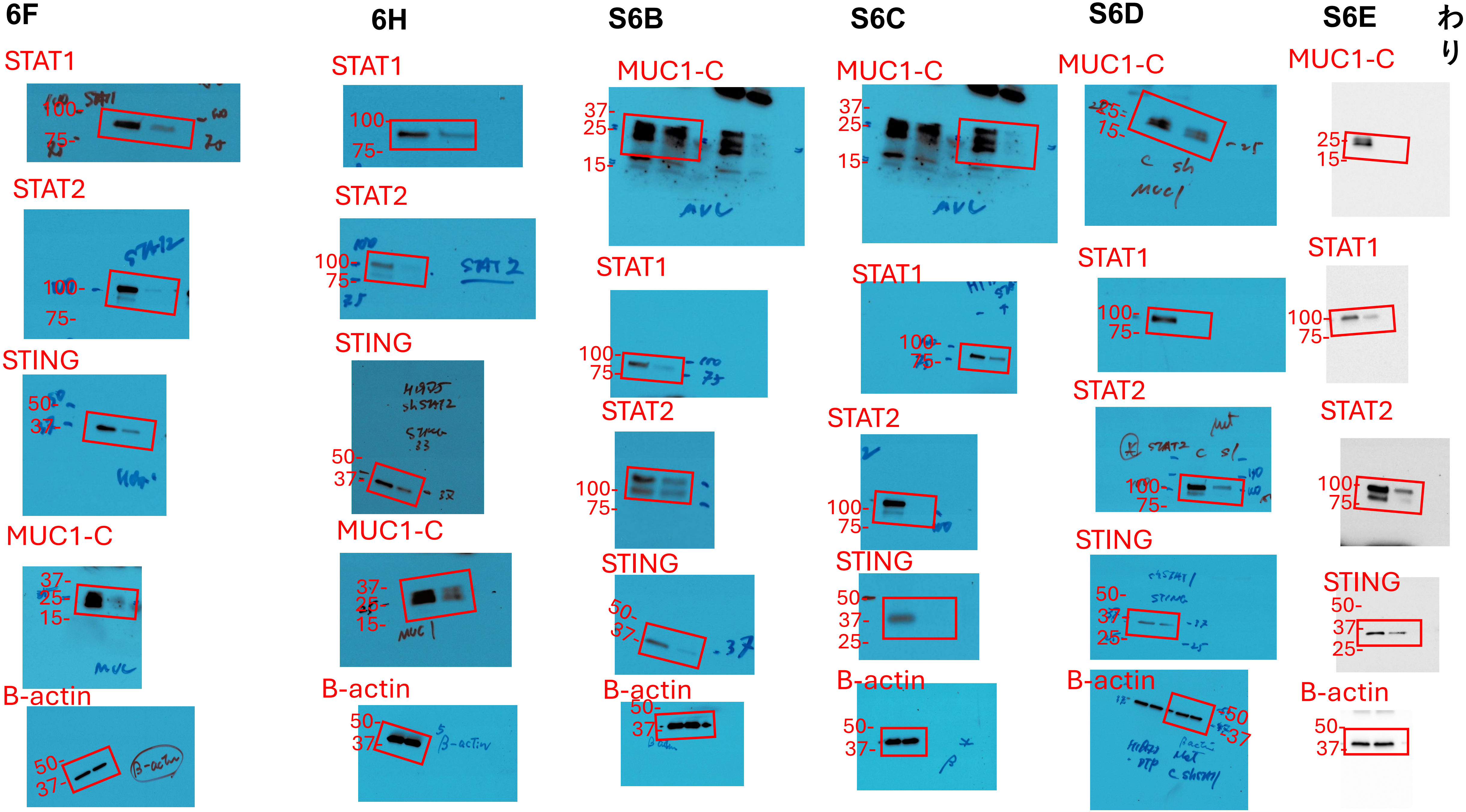
6F
STAT1
STAT2
STING
MUC1-C
B-actin
6H
STAT1
STAT2
STING
MUC1-C
B-actin
S6B
MUC1-C
STAT1
STAT2
STING
B-actin
S6C
MUC1-C
STAT1
STAT2
STING
B-actin
S6D
MUC1-C
STAT1
STAT2
STING
B-actin
S6E
MUC1-C
STAT1
STAT2
STING
B-actin

S6F

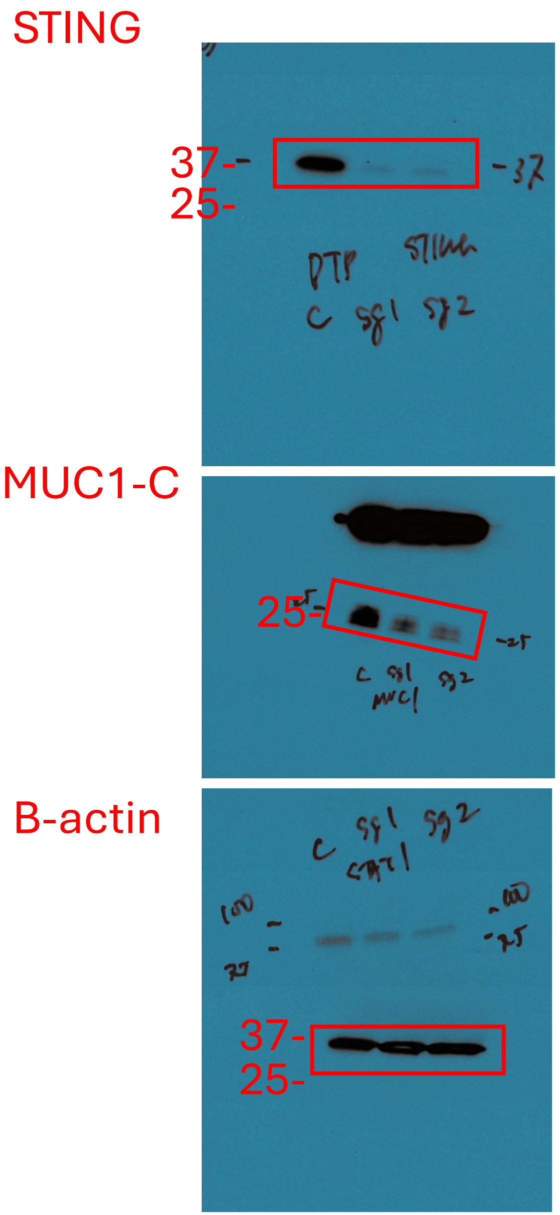
STING
37-
25-
MUC1-C
25-
B-actin
37-
25-

S6G

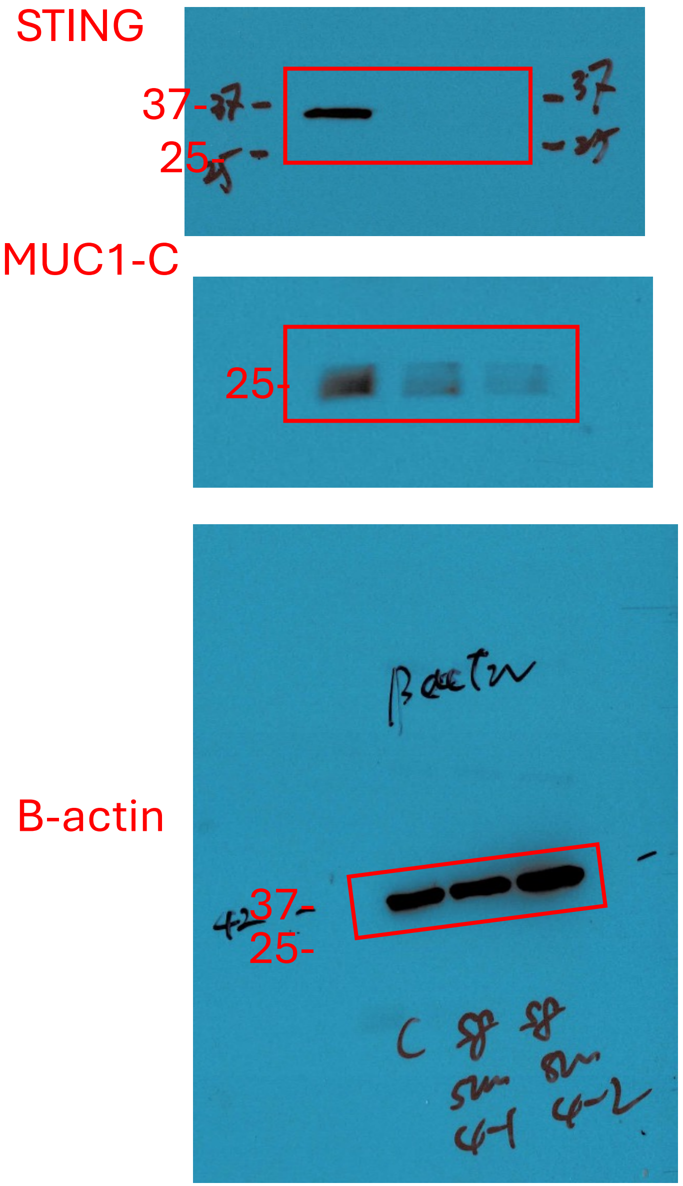
STING
37-
25-
MUC1-C
25-
B-actin
37-
25-
